# Metagenomics-Based, Strain-Level Analysis of *Escherichia coli* From a Time-Series of Microbiome Samples From a Crohn's Disease Patient

**DOI:** 10.3389/fmicb.2018.02559

**Published:** 2018-10-30

**Authors:** Xin Fang, Jonathan M. Monk, Sergey Nurk, Margarita Akseshina, Qiyun Zhu, Christopher Gemmell, Connor Gianetto-Hill, Nelly Leung, Richard Szubin, Jon Sanders, Paul L. Beck, Weizhong Li, William J. Sandborn, Scott D. Gray-Owen, Rob Knight, Emma Allen-Vercoe, Bernhard O. Palsson, Larry Smarr

**Affiliations:** ^1^Department of Bioengineering, University of California, San Diego, La Jolla, CA, United States; ^2^Center for Algorithmic Biotechnology, Institute for Translational Biomedicine, St. Petersburg State University, St. Petersburg, Russia; ^3^St. Petersburg Academic University, Russian Academy of Sciences, St. Petersburg, Russia; ^4^Department of Pediatrics, University of California, San Diego, La Jolla, CA, United States; ^5^Department of Molecular and Cellular Biology, University of Guelph, Guelph, ON, Canada; ^6^Department of Molecular Genetics, University of Toronto, Toronto, ON, Canada; ^7^Division of Gastroenterology, University of Calgary, Calgary, AB, Canada; ^8^Human Longevity Inc., San Diego, CA, United States; ^9^J. Craig Venter Institute, La Jolla, CA, United States; ^10^Department of Medicine, University of California, San Diego, La Jolla, CA, United States; ^11^Inflammatory Bowel Disease Center, University of California, San Diego, La Jolla, CA, United States; ^12^Department of Computer Science and Engineering, University of California, San Diego, La Jolla, CA, United States; ^13^Center for Microbiome Innovation, University of California, San Diego, La Jolla, CA, United States; ^14^The Novo Nordisk Foundation Center for Biosustainability, Technical University of Denmark, Lyngby, Denmark; ^15^California Institute for Telecommunications and Information Technology, University of California, San Diego, La Jolla, CA, United States

**Keywords:** inflammatory bowel disease, *Escherichia coli*, metagenomics, gut microbiome, *de novo* assembly

## Abstract

Dysbiosis of the gut microbiome, including elevated abundance of putative leading bacterial triggers such as *E. coli* in inflammatory bowel disease (IBD) patients, is of great interest. To date, most *E. coli* studies in IBD patients are focused on clinical isolates, overlooking their relative abundances and turnover over time. Metagenomics-based studies, on the other hand, are less focused on strain-level investigations. Here, using recently developed bioinformatic tools, we analyzed the abundance and properties of specific *E. coli* strains in a Crohns disease (CD) patient longitudinally, while also considering the composition of the entire community over time. In this report, we conducted a pilot study on metagenomic-based, strain-level analysis of a time-series of *E. coli* strains in a left-sided CD patient, who exhibited sustained levels of *E. coli* greater than 100X healthy controls. We: (1) mapped out the composition of the gut microbiome over time, particularly the presence of *E. coli* strains, and found that the abundance and dominance of specific *E. coli* strains in the community varied over time; (2) performed strain-level *de novo* assemblies of seven dominant *E. coli* strains, and illustrated disparity between these strains in both phylogenetic origin and genomic content; (3) observed that strain ST1 (recovered during peak inflammation) is highly similar to known pathogenic AIEC strains NC101 and LF82 in both virulence factors and metabolic functions, while other strains (ST2-ST7) that were collected during more stable states displayed diverse characteristics; (4) isolated, sequenced, experimentally characterized ST1, and confirmed the accuracy of the *de novo* assembly; and (5) assessed growth capability of ST1 with a newly reconstructed genome-scale metabolic model of the strain, and showed its potential to use substrates found abundantly in the human gut to outcompete other microbes. In conclusion, inflammation status (assessed by the blood C-reactive protein and stool calprotectin) is likely correlated with the abundance of a subgroup of *E. coli* strains with specific traits. Therefore, strain-level time-series analysis of dominant *E. coli* strains in a CD patient is highly informative, and motivates a study of a larger cohort of IBD patients.

## 1. Introduction

Dysbiosis of the gut microbiome in inflammatory bowel disease (IBD) patients is associated with reduced bacterial diversity, an increase in relative abundance of Proteobacteria (Mukhopadhya et al., 2012), and decline in Firmicutes (Matsuoka and Kanai, 2015). Specifically, *E. coli* is considered one of the potential causes of IBD formation and progression (Rhodes, 2007; Sasaki et al., 2007). One specific pathotype, adherent-invasive *E. coli* (AIEC), which is able to attach to intestinal epithelial cells and survive and replicate within macrophages, has been implicated in intestinal inflammation (Darfeuille-Michaud et al., 1998; Palmela et al., 2017). Members of this pathotype, as well as other IBD-associated *E. coli* isolates, mainly belong to phylogroup B2 (Petersen et al., 2009), carrying a diverse set of virulence factors and displaying distinct metabolic phenotypes (Martinez-Medina and Garcia-Gil, 2014; Fang et al., 2018). However, no unique genetic determinant has been identified for this group (O'Brien et al., 2016).

Previous studies on *E. coli* in IBD mainly focused on clinical isolates extracted from intestinal biopsy and fecal samples, which are then cultured and experimentally characterized (Eaves-Pyles et al., 2008; Vejborg et al., 2011; Desilets et al., 2016; O'Brien et al., 2016). However, most of these studies did not take into consideration other factors including composition of the gut microbiome and dynamics of the community. Recently, with the drop in sequencing costs, metagenomics data has become a popular source of information with which to investigate the composition (Pascal et al., 2017), function (Morgan et al., 2012; Ni et al., 2017) and dynamics (Halfvarson et al., 2017; Schirmer et al., 2018) of the IBD microbiome. However, these studies lack a detailed characterization of the *E. coli* community. They generally only examine the relative abundance of *E. coli*, overlooking the strain-level composition and strain-specific traits of the *E. coli* community, yet previous study has already showed genetic diversity and temporal variation in the *E. coli* population (Caugant et al., 1981).

Fortunately, strain-level analysis of metagenomics data has been made possible with recently developed bioinformatics tools, including MIDAS, that characterizes strain-level variation (Nayfach et al., 2016), DESMAN, that allows *de novo* extraction of strains (Quince et al., 2017), among other strain-level population genomics tools (Luo et al., 2015; Fischer et al., 2017; Truong et al., 2017). Additionally, tools developed for genome-level analysis, such as genome-scale metabolic models (GEMs), enable comprehensive strain-level analysis. GEMs are reconstructions of the metabolic network of strains that are subsequently converted to computable mathematical models, allowing mapping between the genetic basis and phenotypic metabolic functions (McCloskey et al., 2013). Due to the versatile genomic content of *E. coli* (Rasko et al., 2008), strain-level GEM analysis has proven to be essential and informative (Monk et al., 2013).

Here, we conducted a pilot study on one IBD patient, specifically a patient with left-sided Crohn's disease (CD), and performed metagenomics-based, strain-level analysis of the patients time-series *E. coli* community. We not only examined the composition of the gut microbiome, relative abundance of *E. coli*, and community dynamics, but also performed strain-level analysis to identify, assemble, and characterize the dominant *E. coli* strains at different time points, followed by experimental validation.

## 2. Results and discussion

### 2.1. Time-series stool samples were collected and sequenced for 3 years

**Figure 1:**
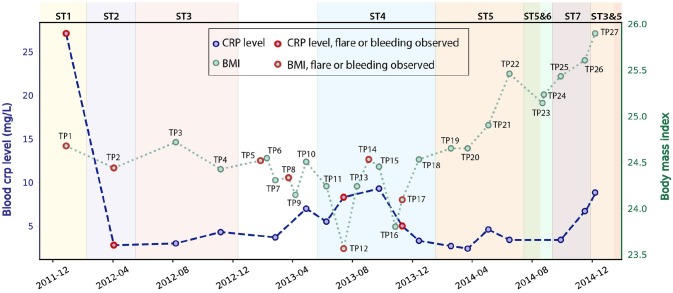
Blood hs-CRP level and BMI of the patient fluctuated during the 3 years of this study (hs-CRP only available for 18 samples). Samples collected during bleeding or flare are labeled in red. The dominant *E. coli* strain varied for different time points (discussed in the next paragraph), and are labeled by different background colors.

We studied 27 time-series stool samples (named TP1-TP27 as shown in Figure 1) collected from a 69 year-old male CD patient, who was diagnosed with colonic CD at the age of 63 with inflammation confined to his sigmoid colon. These samples were collected over a period of 3 years between 2011 and 2014 (Table S1), covering both stable and inflamed states (Wu et al., 2013). We generated metagenomics data for each sample collected, and recorded detailed metadata including body mass index (BMI), blood C-reactive protein (CRP) level, fecal calprotectin level, and other biomarker measurements during this period (Table S1). During the 3 years, this patient took Ciprofloxacin, Metronidazole, and Prednisone daily in February 2012, and also used Lialda and Uceris from June to November 2013. BMI was recorded for all samples and ranged between 23.6 and 25.9 (Figure 1). High-Sensitivity CRP (hs-CRP) level, which is indicative of inflammation level, was measured for 18/27 samples, and fluctuated between 2.4 and 27.1 mg/L (Figure 1). Fecal calprotectin showed a trend similar to blood hs-CRP level, with significant variation (Figure S2). In particular, blood and fecal inflammation levels were the highest when the first sample was collected, with hs-CRP peaked at 27.1 mg/L, while the normal range of hs-CRP for healthy controls is ≤ 1 mg/L (Mosli et al., 2015), and with Calprotectin peaking at 2500, over 50x the upper limit for healthy controls. Therefore, we aimed to explore the relationship between inflammation status and gut microbes, especially with the *E. coli* community in the gut microbiome.

### 2.2. Composition of the gut microbiome and *E. coli* community changed over time

**Figure 2:**
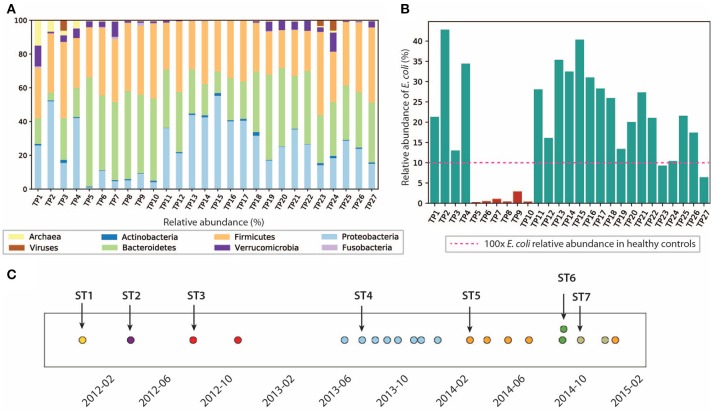
Composition of the gut microbiome and the *E. coli* community is dynamic. **(A)** Relative abundance of microbes at phyla level. **(B)** Relative abundance of *E. coli* in this patient. *E. coli* relative abundance is < 0.1% in the healthy cohort. **(C)** Dominant strains of the *E. coli* community identified in 21/27 samples. Colors represent different dominant strains. Arrows highlight the samples we selected for further analysis on dominant strains.

Analysis of the gut microbiome composition and richness indicates that the gut microbial community of this patient was dysbiotic, and highly dynamic during the 3 years of this study. We performed taxonomy assignment for the metagenomic samples using MetaPhlan2 (Truong et al., 2017), and calculated the alpha and beta diversity of the 27 samples. Compared to the gut microbiome of healthy controls that are mostly dominated by Firmicutes (49–76%) and Bacteroidetes (16–23%) (Matsuoka and Kanai, 2015) with a minor component of Proteobacteria (median = 1%) (Bradley and Pollard, 2017), this patient had an elevated level of Proteobacteria ranging from 1.09 to 55.3%, and a reduced level of Firmicutes between 22.3 and 49.1%. We also found enterobacteria phages K1E (accession: NC_007637.1) and K1-5 (accession: NC_008152.1) in TP1, which are not shown in MetaPhlan2 results in (Figure 2) (see Supplementary Material for detailed analysis). We also performed principal coordinate analysis (PCoA) on the beta diversity calculated (see Figure S3) to evaluate the dissimilarity between samples.

In particular, we characterized the *E. coli* community in the gut microbiome, since *E. coli* is considered one of the leading bacterial triggers in IBD (Rhodes, 2007). The relative abundance of *E. coli* in this patient ranges from 0.1 to 42.6%, which was abnormally high (as much as 400x) compared to that of the healthy controls (≤0.1% in the healthy cohort Human Microbiome Project Consortium, 2012, but consistent with elevated *E. coli* abundance observed in previous IBD studies (Matsuoka and Kanai, 2015). During the 3 years of study, the *E. coli* level remained relatively high, except for the first 4 months of 2013, during which TP5-TP10 were collected (highlighted in red in Figure 2B). The inflammation level during this particular period did not show significant differences compared to other time points. Interestingly, the relative abundance of *E. coli* did not necessarily correlate with inflammation level in all samples. For example, TP2 has the highest *E. coli* relative abundance of 42.6%, yet it only has a hs-CRP level of 2.8 mg/L (1/10 of the hs-CRP level for TP1). Since *E. coli* is a highly versatile species with an open pan-genome (Snipen et al., 2009), it is possible that only a subset of *E. coli* strains with certain pathogenic features contribute to disease progression in IBD. Therefore, we further investigated the strain-level composition for the *E. coli* community in the 21 samples that have ≥5% *E. coli* relative abundance (highlighted in green in Figure 2B). Six samples (TP5-TP10) were excluded from further *E. coli* studies due to their scarcity of *E. coli*.

Single-nucleotide variants (SNV) analysis on the selected 21 samples suggests that the *E. coli* community was dominated by a single strain in most samples, and the dominant strain switched over time. SNV frequencies for *E. coli* species were detected by MIDAS (Nayfach et al., 2016). Most SNV frequencies are close to 0 or 1 (Figure S4), implying that a single strain was typically dominating the *E. coli* community at a given point in time. This result is consistent with a finding in a previous study that a single strain dominates most species in the gut microbiome (Truong et al., 2017).

Positions of the detected SNVs across multiple samples also suggest that the dominant *E. coli* strain changed over time (see section 5) (Figure 2C), potentially due to alterations in diet, microbiome ecological structure, and environment (including the components of the human immune system). In the 21 samples with higher *E. coli* relative abundance, we identified a total of seven dominant strains (some of them abundant in several time points). To further characterize the dominant strains and understand their association with inflammation, we then focused on the highlighted samples in Figure 2C that contain the seven dominant strains.

### 2.3. Dominant *E. coli* strains assembled and computationally characterized

We attempted to recover genome sequences of the seven dominant *E. coli* strains from the selected samples. Draft assemblies of the dominant strains (named ST1-ST7) were obtained by *de novo* metagenomic assembly and binning of individual samples (see section 5), followed by functional annotation using Prokka (Seemann, 2014). Numbers of protein coding genes in the resulting annotations range from 4, 411 to 5, 213 (Table 1). In addition, we performed phylogenetic analysis using PhyloPhlan (Segata et al., 2013) to infer the phylogroup of each assembly. Although previous studies have shown that strains in B2 and D phylogroups are more frequently found in IBD patients (Kotlowski et al., 2007), the seven dominant strains in this patient have diverse phylogenetic origins and are predicted to span phylogroups B2, E, D, B1, and A. In particular, ST1 and ST5 likely belong to phylogroup B2, which contains most of AIEC strains. In addition, we have also assigned the sequence types of the dominant strains using the *de novo* assemblies and BacWGSTdb (Ruan and Feng, 2016). The dominant strains are reported to have different sequence types (Table 1). Specifically, sequence type 95, 69, and 131 are predominant in extraintestinal pathogenic *E. coli* strains (Doumith et al., 2015).

**Table 1:** Characteristics of the seven dominant strains recovered from metagenomic samples.

**Name**	**Time**	**Number of CDS**	**Inferred phylogroup**	**Sequence type**
ST1	2011/12/28	5, 134	B2	95
ST2	2012/04/03	5, 213	E	1,629
ST3	2012/08/07	4, 591	D	69
ST4	2013/07/14	4, 618	B1	58
ST5	2014/03/23	4, 498	B2	131
ST6	2014/08/25	4, 411	A	409
ST7	2014/09/28	4, 487	B1	1,727

To further explore the diversity of the selected strains, we constructed a pan-genome for the seven assemblies and found significant variation between strains. We built the pan-genome with Roary (Page et al., 2015) using a threshold of 80% for gene similarity (see section 5). We identified a total of 8,459 orthologs, of which only 37.7% are core genes shared between all strains. Among the rest of the accessory genes, 39.9% are unique to only one strain (Figure S5), highlighting the diversity of the seven strains. To further explore the variation between strains, we next investigated the genomic features and metabolic functions of the dominant strains.

### 2.4. The analysis of recovered strains reveals a diversity of virulence factors

We examined the distribution of virulence factors in the seven assemblies. For comparison, we included two well-studied AIEC strains, NC101 (Allen-Vercoe and Jobin, 2014; Ellermann et al., 2015), associated with inducing colon-cancer (Arthur et al., 2012), and LF82, an *E. coli* strain associated with right-sided ileal CD patients (Darfeuille-Michaud, 2002; Darfeuille-Michaud et al., 2004; Miquel et al., 2010). In addition, we included the widely studied commensal strain K-12 MG1655 as a well-defined reference strain. We first mapped the seven genome assemblies and three reference strains to a curated virulence factor database VFDB (Chen et al., 2005) using BLAST (Boratyn et al., 2012) with a threshold of 80% sequence similarity. This procedure identified a total of 164 virulence factors amongst the ten strains (Figure S6). Many of these virulence factors are involved in functions that are previously implicated in pathophysiology in IBD, including iron-acquisition (Dogan et al., 2014), adhesion (Barnich et al., 2007), secretion systems (Nash et al., 2010), and capsule synthesis (Martinez-Medina et al., 2009). We observed that strains in phylogroup B2 (NC101, LF82, ST1, ST5) generally have more virulence factors compared to the other strains, and have more virulence factors in common.

### 2.5. Presence/absence of 57 known IBD-associated virulence factors in the recovered strains

**Figure 3:**
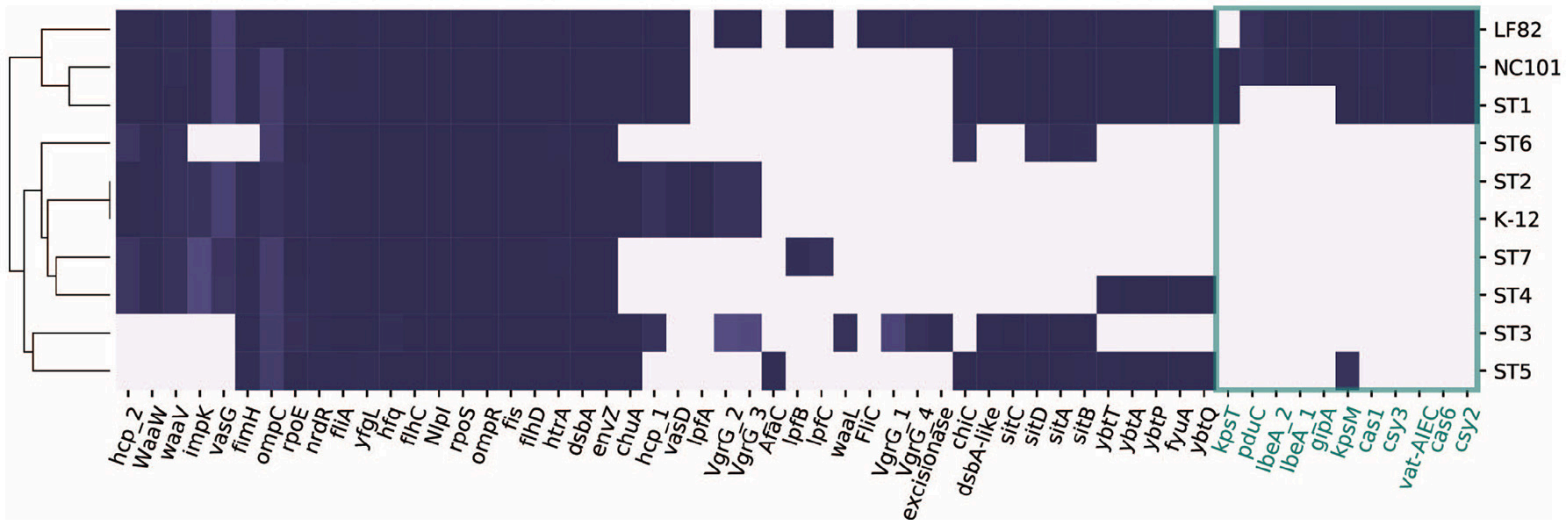
Distribution of 57 genes that were implicated in AIEC pathogenesis in ten strains. Genes unique to ST1, NC101, and LF82 are involved in various functions including capsule synthesis (*kpsT*, Martinez-Medina et al., 2009), mucins protease (*vat-AIEC*, Gibold et al., 2016), CRISPR-associated genes (*cys3, cas6, cys2, and cas1*, Zhang et al., 2015), invasion (*ibeA* and its variant, Cieza et al., 2015), phage encoded VFs (*gipA*, Vazeille et al., 2016), and propanediol utilization (*pduC*, Dogan et al., 2014).

We next focused on 57 genes that have been associated with pathogenicity in IBD patients from previous studies (Table S2). We collected the genes and their sequences from literature, and mapped them against the ten strains using BLAST (Boratyn et al., 2012). Interestingly, only ST1 clustered with the representative AIEC strains LF82 and NC101, while ST5 did not share as many genes with the selected pathogenic strains (Figure 3). This could potentially explain why ST1 correlated with high inflammation level, while hs-CRP was only 2.4 mg/L when ST5 was collected. We found a set of genes that are unique or more prevalent in ST1, LF82, and NC101 that differentiate them from other strains (highlighted in Figure 3). Besides IBD-associated virulence factors, we also found that similar to NC101, ST1 also harbors the polyketide synthase (*pks*) genotoxic island that was shown to induce colorectal cancer (Arthur et al., 2012).

### 2.6. Metabolic networks differentiate ST1 and AIEC strains from other dominant strains collected during periods of low inflammation

Besides virulence factors, we also delineated the differences in metabolism between strains. We built draft metabolic networks for seven assemblies and the three reference strains based on the previously published multi-strain genome-scale metabolic models (GEMs) (Monk et al., 2013) (see section 5). For the ten metabolic networks reconstructed, there are 3,077 metabolic reactions in total, among which 302 are accessory reactions missing from at least one strain, and 2,775 core reactions that are present in all strains.

**Figure 4:**
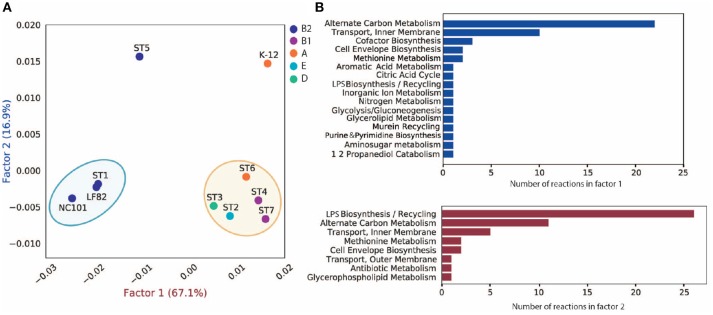
MCA analysis of pan-reactome for ten strains. **(A)** Visualization of factor 1 and factor 2 of MCA results. **(B)** Functional distribution of important reactions in factor 1 and factor 2.

To investigate the discrepancy in metabolic functions between these strains, we created a pan-reactome for these ten strains (see section 5). We then performed multiple correspondence analysis (MCA) on the pan-reactome matrix formed by absence/presence calls for these reactions, which has been shown to effectively classify reactomes (Monk et al., 2014). We then focused on factor 1 and factor 2 (Figure 4A), since they explained a total of 84% variance (67.1 and 16.9%, respectively).

The plot of factor 1 vs. factor 2 (Figure 4A) shows that TP1 is very similar to NC101 and LF82 in terms of metabolic functions, while strains isolated from other time points displayed diverse characteristics (Figure 4A). We observed that factor 1 separated B2 strains from non-B2 strains, while factor 2 separated TP5 and K12 from the other strains. We further investigated the 50 reactions that have the greatest contribution to factor 1 and 2 (Table S3), and plotted their functional distribution (Figure 4B). Many of the top contributing reactions in factor 1 are involved in alternative carbon metabolism, cofactor biosynthesis, and transport reactions. Further analysis showed B2 and non-B2 strains have distinct reactions involved in carbon utilization and metabolite transport (Figure S7A), suggesting that B2 strains and non-B2 strains may be adapted to different microenvironments and nutrient substrates.

For the top contributing reactions in factor 2, although some are also involved in carbon metabolism and transport reactions, more than half of the reactions are engaged in lipopolysaccharide (LPS) biosynthesis and recycling. Additional analysis showed that TP5 and K12 have a unique set of reactions involved in LPS synthesis compared to the other eight strains (Figure S7B). Previous studies showed that endotoxicity of LPS produced by intestinal microbiota plays a vital role in the development of intestinal colitis (Gronbach et al., 2014). Thus, the difference we observed in LPS biosynthesis may correlate with host inflammation status, and needs to be experimentally studied in the future.

MCA analysis of the pan-reactome showed similarity in metabolic functions between ST1 and AIEC strains LF82 and NC101, suggesting that *E. coli* strains associated with intestinal inflammation in IBD patients may share certain metabolic capabilities. However, because we only obtained *de novo* assemblies that are incomplete, we could not construct accurate GEMs to further evaluate their growth capabilities. To verify our results and enable accurate GEM simulation of the most interesting ST1 strain, we proceeded with its experimental isolation, sequencing, and characterization.

### 2.7. ST1 isolation and characterization

Since ST1 was present in high abundance during peak inflammation and showed the closest resemblance to known AIEC strains, we proceeded to isolate ST1 from the stool sample and characterize it experimentally. Its identity was confirmed with SNV analysis (see section 5). This strain, which we named CG1MAC was sequenced and assembled to give a 5,169,659 bp genome with 4,916 coding regions, of which 4,905 genes were present in the ST1 assembly. The accuracy of the ST1 assembly, compared to CG1MAC, is 95.5%. Additional genomic analysis showed that CG1MAC is closely-related to 3_2_53FAA (sharing 4837/4916 ORFs), an *E. coli* strain previously isolated from the inflamed left-sided descending colon of a 52-year-old male CD patient, and is part of the HMP reference genome collection with the strain identification number HM-38 (Human Microbiome Jumpstart Reference Strains Consortium et al., 2010) (see Supplementary Material). We note the similarity in gender, age, and colon inflammation site with our patient. Additionally, the serotype of CG1MAC was experimentally determined to be O2:H7 by the National Microbiology Laboratory in Canada. Phylogenetic analyses suggest that CG1MAC is evolutionarily closely related to AIEC and uropathogenic (UPEC) strains in phylogroup B2 (Figure S8).

To examine whether CG1MAC exhibited AIEC characteristics, we conducted adhesion and invasion assays. Experimental results showed that CG1MAC is able to adhere well to the intestinal epithelial cell line Caco-2, but does not invade THP-1 macrophages, unlike the representative AIEC strain LF82. CG1MAC was engulfed at a low level and showed poor survival intracellularly (see Supplementary Material).

### 2.8. Growth capability of CG1MAC is predicted to be similar to that of AIEC strains

We built a draft genome-scale model (GEM) for CG1MAC based on its genome sequence and previously published *E. coli* models (Monk et al., 2013) (see section 5). The GEM for the CG1MAC strain contains 1,581 genes, 2,913 metabolic reactions, and 2,115 metabolites. We then predicted the growth capability of CG1MAC, along with three draft reference models K-12, LF82, and NC101 that were reconstructed following the same procedure.

**Figure 5:**
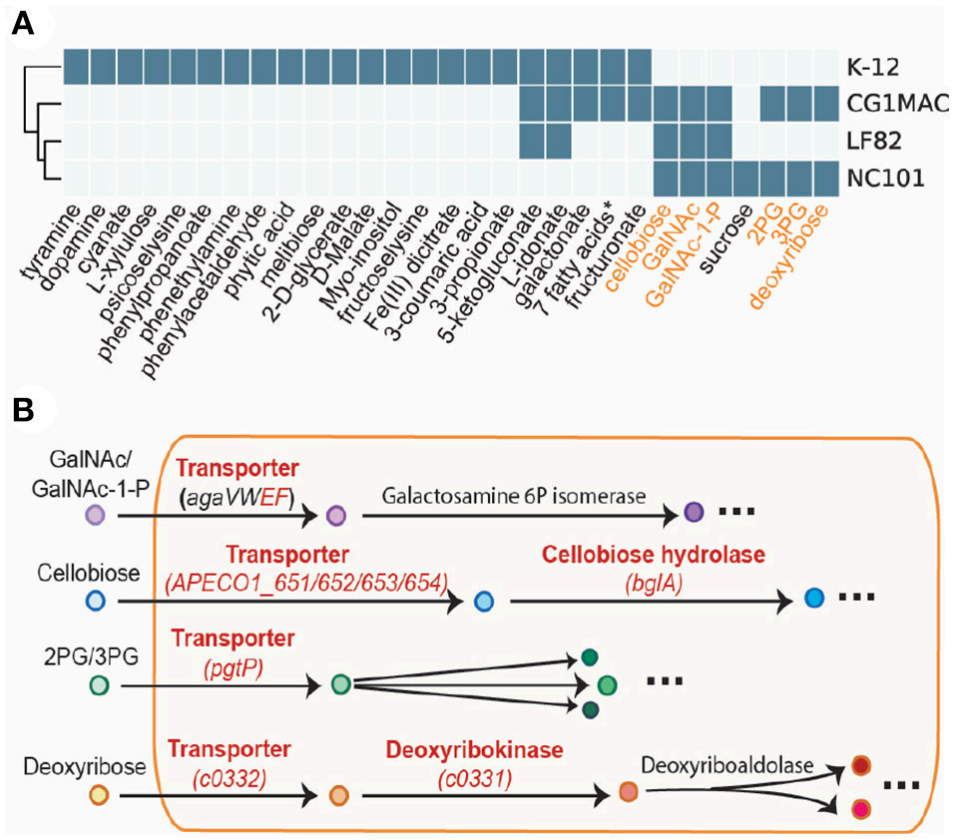
Simulation results of four GEMs. **(A)** Growth capabilities on various nutrient sources can be used to differentiate between strains. **(B)** The key pathways involved in the capability to catabolize the six highlighted substrates. Enzymes in red are missing from *E. coli* K-12.

Growth simulation results on various nutrient sources indicate that CG1MAC is similar to AIEC strains in terms of growth capability. Growth predictions suggest the four strains (CG1MAC, K-12, LF82, and NC101) have distinct metabolic capabilities, as their predicted growth ability differs for 35 substrates (Figure 5A). The predicted growth phenotype displayed by CG1MAC is similar to LF82 and NC101, as they share the ability to utilize a subset of six substrates (labeled in orange in Figure 5A), but not K-12. Among the six identified substrates, some are found abundantly in the intestine, including cellobiose, a derivative of an insoluble dietary fiber cellulose (Cummings, 1984; Cocinero et al., 2009), as well as monosaccharides derived from intestinal mucosa: N-acetyl-D-galactosamine (GalNAc) and N-acetyl-D-galactosamine 1-phosphate (GalNAc 1P) (Ravcheev and Thiele, 2017). The ability to utilize deoxyribose, on the other hand, suggests pathogenicity of CG1MAC and NC101. A previous study showed that the capability to metabolize deoxyribose is associated with the pathogenic potential of intestinal and extraintestinal *E. coli* strains, as this ability increases their competitiveness (Bernier-Fébreau et al., 2004). Deoxyribose availability also promotes host colonization of the intestine by pathogenic *E. coli* strains (Martinez-Jéhanne et al., 2009). The remaining two substrates, 3-phospho-D-glycerate (3PG) and 2-phospho-D-glycerate (2PG), are important intermediates in glycolysis (Neidhardt and Curtiss, 1999), and precursors for amino acid biosynthesis (Kaleta et al., 2013). The ability to directly uptake these substrates potentially enables NC101 and CG1MAC to generate energy more efficiently, thus likely to outcompete other microbes. We have also identified reactions that enable growth on the above six substrates, that are missing from K-12 (labeled in red in Figure 5B). We observed that K-12 lacks transporters for all six substrates, as well as some downstream enzymes. We also performed experimental growth experiments for model validation (see Supplementary Material and Table S4).

## 3. Discussion

In this study we performed metagenomic-based, strain-level analysis of *E. coli* in a time-series of stool samples from a CD patient. The key findings are as follows: (1) The *E. coli* community was highly dynamic in this patient, with different relative abundance and dominant strains at different time points. (2) We were able to extract strain-level *de novo* assemblies of seven dominant strains from metagenomics data, and showed large variation in genomic content among strains using a pan-genome analysis. (3) Comparative genomic analysis and metabolic network reconstruction suggest ST1 (isolated during peak inflammation) resembles known AIEC reference strains NC101 and LF82, while other strains collected during stable states displayed diverse characteristics. (4) To assess the accuracy of *de novo* assemblies from metagenomics data, we isolated ST1 (named CG1MAC) from the stool sample, sequenced and experimentally characterized it. (5) We then built a complete genome-scale metabolic model of CG1MAC and assessed its growth capability.

Detailed time-series data not only showed intestinal dysbiosis of this patient, but also revealed the dynamics of his gut microbiome at strain level. Although recent studies have already shown dramatic fluctuations in both composition and function of the gut microbiome of IBD patients (Halfvarson et al., 2017; Schirmer et al., 2018), and linked it to disease development (Sharpton et al., 2017), they only focused on species level evaluations. In this study, however, we presented strain-level dynamics of the *E. coli* community: not only did relative abundance of *E. coli* vary over time, we also identified seven strains that dominated the *E. coli* community at different time points, which are later shown to have diverse gene contents and phylogenetic origins by *de novo* assemblies.

Strain-level analysis of the dominant *E. coli* strains and their correlation with metadata led us to hypothesize that only certain *E. coli* strains with specific features contribute to IBD progression. Comparative genomic analysis and metabolic network reconstructions suggest similarity in both virulence factors and metabolic functions between ST1 (collected during peak inflammation) and known pathogenic IBD isolates NC101 and LF82. Evidence from literature suggests that the AIEC pathotype, to which both LF82 and NC101 belong, is implicated in IBD. However, we isolated and experimentally characterized ST1 (later named CG1MAC), and found that it does not display AIEC phenotypes. Interestingly, previous studies focused on clinical isolates have also isolated non-AIEC strains from IBD patients, as well as AIEC strains from healthy controls (O'Brien et al., 2016). These results suggest that strains capable of eliciting an inflammatory response in IBD patients may share certain features, but they may not necessarily belong to the AIEC pathotype. Although this result needs to be further verified, both experimentally and in a larger cohort, it illustrates the importance of strain-level evaluations of gut microbiome. Another aspect that needs to be taken into consideration in future study is the association between the strain-specific features and the subtypes of IBD (ileal CD, colonic CD and ulcerative colitis), as research has shown that the three subtypes are genetically determined and may be triggered by different external factors (Cleynen et al., 2016).

Moreover, with the sequence of CG1MAC, we confirmed the validity of the *de novo* assemblies, and characterized the growth capability of CG1MAC with an accurate GEM. Strain-level *de novo* assemblies have not been widely adopted in microbiome studies, but we illustrated the potential and feasibility of such analysis, as the ST1 assembly accurately captures 95.5% of the actual genome content. On the other hand, another powerful tool—GEMs, allowed us to predict that: CG1MAC, along with NC101 and LF82, are able to utilize substrates that are either abundant in the human gut (including cellobiose and mucus glycan), or substrates that potentially enable them to outcompete other strains such as deoxyribose (Bernier-Fébreau et al., 2004; Martinez-Jéhanne et al., 2009).

Additionally, medication also plays an important role in gut microbiome composition. Antibiotics including Ciprofloxacin and Metronidazole have been shown to lower bacterial diversity and decrease abundance of enterobacteria (Langdon et al., 2016), while corticosteroid such as Prednisone and Uceris may contribute to substantial shift in gut microbiota (Huang et al., 2015). Additionally, this patient has also taken mesalamine (Lialda) that has been shown to decrease abundance of Escherichia and Shigella (Morgan et al., 2012). We observe in this patient that after taking Ciprofloxacin, Metronidazole and Prednisone in February 2012, the CRP level dropped dramatically, while the alpha diversity also decreased (Figure S3). After taking Uceris and Liada in 2013 from June to November, no more bleeding or flare was observed. However, more data and experiments are needed to obtain a comprehensive understanding of the impact of medications on microbiome structure and disease progression.

We also recognize some limitations of this approach that need to be addressed going forward: (1) The accuracy of *de novo* assembly at strain-level from metagenomics data needs to be carefully evaluated. Our study showed that such assembly does not capture the genome sequence at 100% accuracy, and such analysis is only possible for samples with high read coverage of *E. coli*. However, with metagenomics analytics tools being rapidly developed, the quality and feasibility of *de novo* assembly at the strain level are expected to be improved in the future. (2) We only examined metagenomics data, not gene expression level in this study. By including metatranscriptomics in the future, one should be able to describe functional states of microbes more accurately. (3) This workflow only allow us to examine the dominant strains at each time point, while *E. coli* strains of lower abundance are not taken into consideration. Therefore, genetic variation in the *E. coli* community at each time point is not characterized. (4) Other factors that contribute to IBD need to be taken into consideration. Association between characteristics of *E. coli* strains and other elements such as host genomics, diet, and their microbial neighbors will likely add valuable insights to future analyses. Overall, we believe performing such an analysis on a large cohort of IBD patients will greatly enrich our knowledge of IBD and the gut microbiome.

## 4. Conclusions

In this study, we observed the dominant *E. coli* strain in this patient varied over time. Particularly, the dominant strains isolated during peak inflammation is most similar to known pathogenic strains implicated in IBD, while other strains collected during more stable states have diverse properties. Overall, this pilot study illustrates that a strain-level analysis of *E. coli* from a time-series of stool samples can be very productive. The approach we utilized in this study not only captures the structure and dynamics of the entire microbiome community, but also allows a detailed evaluation of *E. coli* at the strain level. Due to decreasing sequencing cost, and fewer experimental procedures involved, this approach should also enable rapid and large-scale analyses in the future.

## 5. Materials and methods

### 5.1. Metagenomics data generation

DNA was extracted from primary fecal samples using the MoBio PowerMag extraction kit (Qiagen Inc). Shotgun metagenomic libraries were prepared and sequenced at the sequencing core facility at the Institute for Genomic Medicine at UCSD. Briefly, libraries were constructed from each sample using 200 ng of extracted DNA, sheared to a target fragment size of approximately 400 bp using a Covaris E220 sonicator, and input to the TruSeq Nano PCR-based library prep kit (Illumina Inc), with samples individually indexed using dual 8 bp barcoded adapters. Amplified libraries were then pooled and sequenced on a HiSeq4000 instrument. Sequenced reads were trimmed of adapter sequences and quality-filtered using Skewer (Jiang et al., 2014) (end-quality trimming parameter of Phred 15 and a minimum length setting of 100 bp after trimming) and cutadapt (Martin, 2011) v1.15 (parameters -m 36 -q 20 -a ATCGGAAGAGCACACGTCTGAACTCCAGTCAC, -A ATCGGAAGAGCGTCGTGTAGGGAAAGAGTGT). Trimmed sequences were then filtered of human-derived reads using Bowtie2 (Langmead and Salzberg, 2012) under the “very-sensitive” setting, only retaining read pairs for which neither pair mapped to the human reference.

### 5.2. Metagenomics data analysis

Taxonomic profiles of the metagenomics data were evaluated using MetaPhlan2 (Truong et al., 2017) with default parameters. We extracted *E. coli* relative abundance from the result and compared it across samples. In addition to MetaPhlan2 analysis, we also performed additional analysis to confirm the presence of bacteriophages in a sample using Bowtie2 by mapping sequencing reads to genome sequences of the two phages (Langmead2012-zv) (see Supplementary Material).

Alpha and beta diversity of the metagenomics data were calculated with the python package skbio (sci, 2018). We utilized the previously calculated taxonomy profiles at species level as the input OTU tables for diversity calculation. We calculated alpha diversity using the metric “observed_otus” and beta diversity using the metric “braycurtis”. We then performed principal coordinate analysis, and plotted the PC1, 2, 3 using the same python package skbio.

### 5.3. Characterization of the dominant *E. coli* strains using single nucleotide variant (SNV) frequencies

First, MIDAS pipeline (database v 1.2) (Nayfach et al., 2016) was used with defaults to call genome-wide SNVs for all abundant species within individual samples. SNVs frequencies information for *E. coli* 58110 (representative genome for *E. coli* species in the MIDAS database) was merged across samples. Figure S4 illustrates minor allele frequencies at particular genomic sites across all samples (positions chosen by MIDAS, columns reordered with respect to their hierarchical clustering). The heatmap suggests that *E. coli* population within most of metagenomic samples was dominated by single strain (only values close to 0 or 1 are observed in respective rows) that changed over time.

We then performed refined computational analysis of the SNV frequencies to confirm this hypothesis and identify samples with the same dominant *E. coli* strain. For each metagenomic sample, MIDAS pipeline with parameter “–species_id Escherichia_coli_58110” was used to compute per-base coverage and SNV frequencies for the *E. coli* reference. To avoid various artifacts, we then discarded sites with aberrant coverage as follows: positions with coverage 0, positions with coverage less than twice the median across the remaining sites, and positions with coverage falling within low/high 10% of the coverage values across the remaining sites.

To test whether the *E. coli* population within the sample is dominated by a single strain we then analyzed variant allele frequencies at the remaining positions. Specifically, we considered population as dominated if less than 0.05% of the positions had a minor variant frequency exceeding 10%. All but two samples (TP23 and TP27) satisfied this condition.

We further attempted to divide the remaining 19 samples into groups dominated by the same strain. We define the similarity between the pair of samples as a fraction of positions in which the major variants matched (only the sites retained in the analysis of both samples were considered). Single-linkage clustering with a 99.9% threshold was used to obtain 7 groups of samples each corresponding to a particular *E. coli* strain. A single sample has been chosen within each group to attempt the reconstruction of the strain genome via *de novo* assembly (see section 5.4).

### 5.4. Assembling dominant *E. coli* strains from metagenomics data

metaSPAdes assembler v3.11.1 with default parameters has been used to perform *de novo* assembly of 7 individual metagenomic samples (12/28/2011; 4/3/2012; 8/7/2012; 7/14/2013; 3/23/2014; 8/25/2014; 9/28/2014).

Resulting scaffolds and their coverage depths (average 56-mer coverage reported by metaSPAdes) were provided as input to MaxBin2 (Wu et al., 2016). Each sample contained a bin annotated as *E. coli* with an estimated completeness exceeding 97% (as reported by CheckM Parks et al., 2015), which was used as a draft assembly for the downstream analysis. We have also considered including smaller bins annotated as *E. coli* by MaxBin2, but it has resulted in sharp increase of the contamination level (as reported by CheckM). Contamination and completeness scores of these assemblies are reported in Table S5.

### 5.5. Phylogenetic analysis and pan-genome construction of the seven assemblies

We first annotated the assemblies using Prokka (Seemann, 2014) with default parameters. The output files from Prokka were then used to perform phylogenetics analysis and pan-genome reconstruction. To perform phylogenetic analysis using PhyloPhlan (Segata et al., 2013), we utilized the protein FASTA files ending in “.faa” from Prokka output, and constructed the phylogenetic trees with 110 other *E. coli* strains with known phylogroups to infer phylogroup of each assembly. To construct pan-genome of the seven assemblies, we used Roary (Page et al., 2015) that takes input files ending in “.gff”, which contain the master annotation in GFF3 format produced by Prokka. We set the parameter “minimum percentage identity for blastp” to 80.

### 5.6. Virulence factor analysis

We mapped genome assemblies of the dominant strains against two sets of virulence factor references. The first set is the curated virulence factors collected from the VFDB database (Chen et al., 2005). The second set is 57 genes identified from literature that are associated with AIEC strains, which are implicated in IBD. These genes are mainly identified and collected according to the review paper by Palmela et al. (2017). Note that the 57 genes contain variants of genes that perform the same functions. We used BLAST (Boratyn et al., 2012) to map the assemblies to the references and considered genes to be present when the sequence similarity is greater than 80%.

### 5.7. Metabolic network reconstruction and pan-reactome matrix analysis

The draft metabolic reconstructions of *E. coli* strains are created based on a previous multi-strain *E. coli* study (Monk et al., 2013). We first created an *E. coli* pan model that combines all the genes, reactions, and metabolite in the 55 *E. coli* models reconstructed by Monk et al. (2013). To incorporate the most recent update in *E. coli* reconstruction, we also added the content of the latest K-12 model iML1515 (Monk et al., 2017) to the pan model. Since all included *E. coli* strains span various pathotypes and phylogenetic origins, the pan model created is considered a comprehensive representation of metabolic functions in *E. coli* strains, as well as a good starting point for metabolic network reconstruction. We then mapped the sequences of strains of interest to all the genes in the pan model using BLAST (McGinnis and Madden, 2004), and set a threshold of 80% for both gene similarity and alignment length, in order for a gene to be considered present in the strains. The missing genes and their correlated reactions and metabolites in each strain are removed from the pan model to create strain-specific metabolic network reconstructions. The metabolic network was reconstructed using the python package COBRApy (Ebrahim et al., 2013).

To compare the metabolic networks of the 7 dominant strains and 3 reference strains, we then created a binary matrix of size 10 by 3,077 that records the presence and absence of each reaction in all 10 strains. To determine the similarity in metabolic functions in 10 strains, we performed MCA analysis using python package mca (mca, 2018) with Benzecri correction, with the parameter of TOL set to 1e-9. To extract the important reactions in factor 1 and factor 2, we identified the top 50 reactions that has the highest contribution to these two factors (Table S3).

### 5.8. Isolation of bacterial strains: CG1MAC and 3_2_53FAA

In order to isolate CG1MAC from the stool sample, we diluted the sample in saline and plated dilutions on McConkey agar to select for *E. coli* isolates. All obtained isolates were picked, and gDNA was extracted using a Qiagen stool mini kit. To verify the isolates that identified with the predicted genotype of the target strain, four genes were used, *fyuA, vasD, xerD, gsp*, to which we designed PCR primers based on sequence data from the *de novo* assembly of ST1. Comparative analysis showed that these genes were present in the metagenomic dataset obtained from the originating stool sample, and are more prevalent in IBD-associated *E. coli* strains. There were 40 strains obtained and screened by PCR in this way, and all were found to positively identify with the ST1 assembly. Of the clones, one was selected for further analysis, and named CG1MAC.

Strain 3_2_53FAA was isolated from an inflamed biopsy sample from the descending colon of a 52 year old male left-sided CD patient in a Calgary, Canada clinic in 2007. The patient had an initial diagnosis of ulcerative colitis which was later changed to Crohn's colitis (ileal biopsies were normal). Strain 3_2_53FAA was placed into the Human Microbiome Project reference genome collection as HM-38, and as such was genome sequenced by the Broad Institute (GenBank assembly accession number GCA_000157115.2).

Both CG1MAC and 3_2_53FAA were serotyped by The National Microbiology Laboratory (Public Health Agency of Canada) at Guelph, Ontario.

### 5.9. Bacterial genome sequence

We sequenced the genome of isolated *E. coli* strain CG1MAC. First, we isolated and purified gDNA from pelleted cells using the Macherey-Nagel NucleoSpin Tissue Kit (Catalog number 740952.50) following the manufacturer's protocol, including RNAse treatment. Second, we prepared a genomic DNA library using a KAPA HyperPlus Library Preparation Kit (catalog number KK8514) incorporating dual indices during the PCR amplification step, and checking quality with TapeStation. Eventually we pooled the library and sequenced using the Illumina HIseq 4000 instrument with paired-end and 100/100 reads settings.

We used SPAdes (Bankevich et al., 2012) to assemble the high quality reads with default parameters. The assembled genome has been submitted to NCBI with accession number QLAC00000000.

### 5.10. Confirmation of CG1MAC isolate identity with SNV analysis

We used genome-wide single nucleotide variant (SNV) frequencies analysis to verify that: (1) the population of *E. coli* in the TP1 metagenomic sample is dominated by a single subpopulation; (2) Dominant subpopulation is represented by isolated CG1MAC strain.

Both TP1 and CG1MAC isolate reads were processed by MIDAS pipeline (Nayfach et al., 2016) with parameter –species_id Escherichia_coli_58110 to compute coverage and SNV frequencies for metagenomic and isolate sequencing reads against *E. coli* reference included in its database.

First we demonstrate that *E. coli* population in TP1 is likely dominated by a single strain. To avoid various artifacts we ignored positions with coverage falling within low/high 10% of the coverage values across all covered positions of the reference. Out of 2.85 million remaining sites only 181 had major allele frequency (MAF) not exceeding 90% (in comparison, CG1MAC sample had 96 of such positions), suggesting that a single strain accounted for the lions share of *E. coli* population. Then we compared the predicted genotypes of the CG1MAC isolate and dominant *E. coli* strain in TP1. Only sites with MAF ≥ 90% and coverage falling within 10 and 90th percentiles in both samples were considered. While they cover 59% of the reference genome (total 2.48Mb), no differences were observed between the major alleles of the two samples, reliably indicating that the CG1MAC isolate originates from the dominant subpopulation.

### 5.11. Curation of the CG1MAC model

First, we created the draft metabolic reconstruction of CG1MAC following the procedure described in section 5.6. We then performed additional curation to improve the accuracy of the draft model. We annotated the genome of CG1MAC with RAST (Aziz et al., 2008) and identified metabolic genes using Enzyme Commision (EC) numbers. We then identified 413 metabolic genes not included in the pan model, and looked into the reactions associated with them in Uniprot database (The UniProt Consortium, 2017) regarding their annotation score and experimental evidence. Among all identified reactions, we only added six to the model based on the following filtering criteria: (1) Have a complete EC number with four numbers; (2) Not involved in DNA/RNA modification, as suggested by the established GEM reconstruction protocol (Thiele and Palsson, 2010); (3) experimentally proven to be present in *E. coli*; (4) have a defined reaction with specificity; (5) do not duplicate with existing reactions. The majority of the identified reactions are already present in the model, as their encoding genes are variants of existing genes in the model. We then added the new reactions to the CG1MAC model and the 3 reference models whenever appropriate, to ensure the growth simulation performed on these four models is accurate. Finally, we performed the manual curation step for CG1MAC model following the established protocol (Thiele and Palsson, 2010). Because the 55 existing models that the reconstruction was based on were already manually curated, we focused on curating newly added reaction. We removed reactions and metabolites in the wrong compartment, added in subsystem of new reactions, ensured the new reactions were mass/charge balanced, and checked gene-protein-reaction (gpr) of newly-added reactions.

### 5.12. Adhesion and invasion assays on Caco-2 and THP-1 cells

To determine bacterial invasion in epithelial cells and survival in macrophages Caco-2, cells were maintained in DMEM + 10% FBS (Invitrogen). THP-1 cells were maintained in RPMI + 10% FBS (Invitrogen) in 5% CO2 humidified atmosphere at 37°C. Differentiation of THP-1 cells was achieved by treatment with 5ng/ml of PMA (Sigma-Aldrich) for 2 days. Cells were allowed to recuperate in normal media for 1 day before assay was performed.

Adhesion, invasion and survival assay were performed as described in Negroni et al. (2012). Briefly, cell invasion analyses were carried out in Caco-2 cells cultured in DMEM without antibiotics, and maintained in 5% *CO*_2_ and 37°C. Cell monolayers were infected with *E. coli* strains at multiplicity of infection (MOI) of 100, for 2h at 37°C. After the infection period, cells were washed with 3 x PBS and placed in fresh medium supplemented with gentamicin (50 μg/ml), incubated for 1 h at 37°C, and lysed with 0.1% Triton-X-100PBS. Lysate serial dilutions were plated on LB agar (Invitrogen) and incubated at 37°C overnight. Cell adhesion analysis was also carried out in Caco-2 cells using similar infection conditions as described for invasion assays, but omitting the gentamicin treatment. Differentiated THP-1 cells were infected with *E. coli* strains (MOI = 100) for 2h at 37°C. Cells were then washed in PBS and placed in fresh medium supplemented with gentamicin (50 μg/ml). Intracellular bacterial content was determined at 1 and 24 h post infection at 37°C and the ratio between bacterial content at each period was determined.

### 5.13. *In silico* growth simulations

Growth simulation for CG1MAC, K-12, LF82 and NC101 were performed using COBRApy. We simulated growth in M9 minimal media, with the lower bound of exchange reactions for the following substrate set to -1000: *Ca*^2+^, *Cl*^−^, *CO*^2^, *Co*^2+^, *Cu*^2+^, *Fe*^2+^, *Fe*^3+^, *H*^+^, *H*_2_O, *K*^+^, *Mg*^2+^, *Mn*^2+^, $Mo{O_{4}}^{2}$, *Na*^+^, *Ni*^2+^, $Se{O_{4}}^{2}-$, $Se{O_{3}}^{2}+$, and *Zn*^2+^. Moreover, the default carbon, nitrogen, sulfur and phosphate sources are glucose, $N{H_{4}}^{-}$, $S{O_{4}}^{2}$, $HP{O_{4}}^{2}$. These reactions have lower bounds set to -1000. Another essential substrate is cob(I)alamin, for which the exchange reaction has a lower bound of −0.01. We evaluated if sole carbon, nitrogen, sulfur or phosphate substrate supported growth. To do so, we set the lower bound of the exchange reaction of the default substrate to 0, and added sole substrate by setting the lower bound of exchange reaction to −10. Additionally, we have simulated growth under aerobic condition by setting the lower bound of oxygen uptake to −10.

### 5.14. Growth experiments

The *E. coli* strains K12 and CG1MAC were grown in modified M9 media with the main carbon, nitrogen, or sulfur source replaced. For tests involving the replacement of the carbon source, glucose was omitted from the M9 media and 0.022 moles/L of the new carbon source was added in its place. For the nitrogen source replacement tests, *NH*_4_*Cl* was replaced with 0.019 moles/L of the new nitrogen source and for the sulfur source replacement tests, *HPO*_4_ • 7 *H*_2_*O* was replaced with 0.001 moles/L of the new sulfur source. For the media used in both the nitrogen and sulfur replacement tests, the glucose concentration was increased to 0.004 g/mL.

Freshly-cultured single colonies of each strain were selected after overnight incubation on blood agar plates and individually diluted in 5 mL of basal M9 media (no carbon, nitrogen or sulfur source) and 100 μL of each diluted strain was used to inoculate 5 mL of modified M9 medium containing the test carbon, nitrogen, or sulfur source. Inoculated tubes were incubated for 24 h at 37°C with orbital shaking at 200 rpm to pre-expose cells to each metabolite. Following incubation, cells were pelleted at 5,000 rpm for 10 min and resuspended in 200 μL PBS buffer, whereupon the optical density at 555 nm was recorded. This value was used to normalize the amount of each culture that was added to 5 mL of the appropriate test medium in a glass test tube. Sample test tubes were incubated for 48 h at 37°C with orbital shaking at 200 rpm, and 100 μL samples of these tubes were used to measure the optical density at 555 nm which was recorded every 24 h using a Wallac Victor 3 plate reader.

Metabolites used were: glucose (Fisher Scientific); melibiose, phenylacetaldehyde, trans-3-Hydroxycinnamic acid, oxaloacetic acid, dopamine hydrochloride, 2-Deoxy-D-ribose, iron (III) citrate, taurine, threonine and sodium thiosulfate (from Sigma Aldrich); cellobiose and D-arabinose (from Fluka); 3-(3-hydroxy-phenyl) propionate, D-arabinose, choline chloride, D-(+)-galactose, 3-hydroxyphenylacetic acid, tyramine and methyl-4-hydroxyphenylacetate (from Alfa Aesar), and sucrose (from Bioshop).

### 5.15. Ethics statement

Patient that had the stool samples collected is consented under two protocols: HRPP #141853 American Gut Project and HRPP #150275 Evaluating the Human Microbiome. Both protocols were approved by University of California San Diegos Human Research Protection Program (HRPP). Written informed consent on dissemination of the result and scientific publication are also included in the approved protocols, and was obtained from the patient.

The patient in which strain 3_2_53FAA was isolated was recruited and consented through the Intestinal Inflammation Tissue Bank at University of Calgary and this study was approved through the Conjoint Health Research Ethics Board of the University of Calgary (Project Numbers; REB14-2429 and REB14-2430).

## Data availability statement

The metagenomics sequence of the 27 samples have been submitted to EBI under study PRJEB24161. Note that these samples are only a subset of the metagenomics data under this study. The genome sequence of CG1MAC can be found in NCBI with accession number QLAC00000000.

## Author contributions

LS, BP, and EA-V designed the study. XF, JM, SN, MA, QZ, JS, and WL performed computational analysis. CG and CG-H performed growth experiments. NL and SG-O performed adhesion/invasion assay. RS generated genome sequence. RK generated metagenomics data. WS and PB generated clinical data, all the authors reviewed and approved the manuscript.

### Conflict of interest statement

The authors declare that the research was conducted in the absence of any commercial or financial relationships that could be construed as a potential conflict of interest.

## References

[B1] Allen-Vercoe E. Jobin C. (2014). Fusobacterium and enterobacteriaceae: important players for CRC? Immunol. Lett. 162(2 Pt A):54–61. 10.1016/j.imlet.2014.05.014 24972311 PMC4259803

[B2] Arthur J. C. Perez-Chanona E. Mühlbauer M. Tomkovich S. Uronis J. M. Fan T.-J.. (2012). Intestinal inflammation targets cancer-inducing activity of the microbiota. Science 338, 120–123. 10.1126/science.1224820 22903521 PMC3645302

[B3] Aziz R. K. Bartels D. Best A. A. DeJongh M. Disz T. Edwards R. A.. (2008). The RAST server: rapid annotations using subsystems technology. BMC Genomics 9:75. 10.1186/1471-2164-9-75 18261238 PMC2265698

[B4] Bankevich A. Nurk S. Antipov D. Gurevich A. A. Dvorkin M. Kulikov A. S.. (2012). SPAdes: a new genome assembly algorithm and its applications to single-cell sequencing. J. Comput. Biol. 19, 455–477. 10.1089/cmb.2012.0021 22506599 PMC3342519

[B5] Barnich N. Carvalho F. A. Glasser A.-L. Darcha C. Jantscheff P. Allez M.. (2007). CEACAM6 acts as a receptor for adherent-invasive *E. coli*, supporting ileal mucosa colonization in crohn disease. J. Clin. Invest. 117, 1566–1574. 10.1172/JCI30504 17525800 PMC1868786

[B6] Bernier-Fébreau C. du Merle L. Turlin E. Labas V. Ordonez J. Gilles A. M.. (2004). Use of deoxyribose by intestinal and extraintestinal pathogenic escherichia coli strains: a metabolic adaptation involved in competitiveness. Infect. Immun. 72, 6151–6156. 10.1128/IAI.72.10.6151-6156.2004 15385522 PMC517565

[B7] Boratyn G. M. Schäffer A. A. Agarwala R. Altschul S. F. Lipman D. J. Madden T. L. (2012). Domain enhanced lookup time accelerated BLAST. Biol. Direct 7:12. 10.1186/1745-6150-7-12 22510480 PMC3438057

[B8] Bradley P. H. Pollard K. S. (2017). Proteobacteria explain significant functional variability in the human gut microbiome. Microbiome 5:36. 10.1186/s40168-017-0244-z 28330508 PMC5363007

[B9] Caugant D. A. Levin B. R. Selander R. K. (1981). Genetic diversity and temporal variation in the *E. coli* population of a human host. Genetics 98, 467–490. 7037535 10.1093/genetics/98.3.467 PMC1214454

[B10] Chen L. Yang J. Yu J. Yao Z. Sun L. Shen Y.. (2005). VFDB: a reference database for bacterial virulence factors. Nucleic Acids Res. 33, D325–D328. 10.1093/nar/gki008 15608208 PMC539962

[B11] Cieza R. J. Hu J. Ross B. N. Sbrana E. Torres A. G. (2015). The IbeA invasin of adherent-invasive *Escherichia coli* mediates interaction with intestinal epithelia and macrophages. Infect. Immun. 83, 1904–1918. 10.1128/IAI.03003-14 25712929 PMC4399045

[B12] Cleynen I. Boucher G. Jostins L. Schumm L. P. Zeissig S. Ahmad T.. (2016). Inherited determinants of crohn's disease and ulcerative colitis phenotypes: a genetic association study. Lancet 387, 156–167. 10.1016/S0140-6736(15)00465-1 26490195 PMC4714968

[B13] Cocinero E. J. Gamblin D. P. Davis B. G. Simons J. P. (2009). The building blocks of cellulose: the intrinsic conformational structures of cellobiose, its epimer, lactose, and their singly hydrated complexes. J. Am. Chem. Soc. 131, 11117–11123. 10.1021/ja903322w 19722675

[B14] Cummings J. H. (1984). Cellulose and the human gut. Gut 25, 805–810. 10.1136/gut.25.8.805 6378732 PMC1432575

[B15] Darfeuille-Michaud A. (2002). Adherent-invasive escherichia coli: a putative new *E. coli* pathotype associated with crohn's disease. Int. J. Med. Microbiol. 292, 185–193. 10.1078/1438-4221-00201 12398209

[B16] Darfeuille-Michaud A. Boudeau J. Bulois P. Neut C. Glasser A.-L. Barnich N.. (2004). High prevalence of adherent-invasive *Escherichia coli* associated with ileal mucosa in crohn's disease. Gastroenterology 127, 412–421. 10.1053/j.gastro.2004.04.061 15300573

[B17] Darfeuille-Michaud A. Neut C. Barnich N. Lederman E. Di Martino P. Desreumaux P.. (1998). Presence of adherent escherichia coli strains in ileal mucosa of patients with crohn's disease. Gastroenterology 115, 1405–1413. 10.1016/S0016-5085(98)70019-8 9834268

[B18] Desilets M. Deng X. Deng X. Rao C. Ensminger A. W. Krause D. O.. (2016). Genome-based definition of an inflammatory bowel disease-associated Adherent-Invasive *Escherichia coli* pathovar. Inflamm. Bowel Dis. 22, 1–12. 10.1097/MIB.0000000000000574 26444104

[B19] Dogan B. Suzuki H. Herlekar D. Sartor R. B. Campbell B. J. Roberts C. L.. (2014). Inflammation-associated adherent-invasive *Escherichia coli* are enriched in pathways for use of propanediol and iron and m-cell translocation. Inflamm. Bowel Dis. 20, 1919–1932. 10.1097/MIB.0000000000000183 25230163

[B20] Doumith M. Day M. Ciesielczuk H. Hope R. Underwood A. Reynolds R.. (2015). Rapid identification of major *Escherichia coli* sequence types causing urinary tract and bloodstream infections. J. Clin. Microbiol. 53, 160–166. 10.1128/JCM.02562-14 25355761 PMC4290915

[B21] Eaves-Pyles T. Allen C. A. Taormina J. Swidsinski A. Tutt C. B. Jezek G. E.. (2008). *Escherichia coli* isolated from a crohn's disease patient adheres, invades, and induces inflammatory responses in polarized intestinal epithelial cells. Int. J. Med. Microbiol. 298, 397–409. 10.1016/j.ijmm.2007.05.011 17900983

[B22] Ebrahim A. Lerman J. A. Palsson B. O. Hyduke D. R. (2013). COBRApy: COnstraints-based reconstruction and analysis for python. BMC Syst. Biol. 7:74. 10.1186/1752-0509-7-74 23927696 PMC3751080

[B23] Ellermann M. Huh E. Y. Liu B. Carroll I. M. Tamayo R. Sartor R. B. (2015). Adherent-Invasive escherichia coli production of cellulose influences Iron-Induced bacterial aggregation, phagocytosis, and induction of colitis. Infect. Immun. 83, 4068–4080. 10.1128/IAI.00904-15 26216423 PMC4567620

[B24] Fang X. Monk J. M. Mih N. Du B. Sastry A. V. Kavvas E.. (2018). *Escherichia coli* B2 strains prevalent in inflammatory bowel disease patients have distinct metabolic capabilities that enable colonization of intestinal mucosa. BMC Syst. Biol. 12:66. 10.1186/s12918-018-0587-5 29890970 PMC5996543

[B25] Fischer M. Strauch B. Renard B. Y. (2017). Abundance estimation and differential testing on strain level in metagenomics data. Bioinformatics 33, i124–i132. 10.1093/bioinformatics/btx237 28881972 PMC5870649

[B26] Gibold L. Garenaux E. Dalmasso G. Gallucci C. Cia D. Mottet-Auselo B.. (2016). The Vat-AIEC protease promotes crossing of the intestinal mucus layer by crohn's disease-associated *Escherichia coli*. Cell. Microbiol. 18, 617–631. 10.1111/cmi.12539 26499863

[B27] Gronbach K. Flade I. Holst O. Lindner B. Ruscheweyh H. J. Wittmann A.. (2014). Endotoxicity of lipopolysaccharide as a determinant of t-cell-mediated colitis induction in mice. Gastroenterology 146, 765–775. 10.1053/j.gastro.2013.11.033 24269927

[B28] Halfvarson J. Brislawn C. J. Lamendella R. Vázquez-Baeza Y. Walters W. A. Bramer L. M.. (2017). Dynamics of the human gut microbiome in inflammatory bowel disease. Nat. Microbiol. 2:17004. 10.1038/nmicrobiol.2017.4 28191884 PMC5319707

[B29] Huang E. Y. Inoue T. Leone V. A. Dalal S. Touw K. Wang Y.. (2015). Using corticosteroids to reshape the gut microbiome: implications for inflammatory bowel diseases. Inflamm. Bowel Dis. 21, 963–972. 10.1097/MIB.0000000000000332 25738379 PMC4402247

[B30] Human Microbiome Jumpstart Reference Strains Consortium Nelson K. E. Weinstock G. M. Highlander S. K. Worley K. C. Creasy H. H.. (2010). A catalog of reference genomes from the human microbiome. Science 328, 994–999. 10.1126/science.1183605 20489017 PMC2940224

[B31] Human Microbiome Project Consortium (2012). Structure, function and diversity of the healthy human microbiome. Nature 486, 207–214. 10.1038/nature11234 22699609 PMC3564958

[B32] Jiang H. Lei R. Ding S.-W. Zhu S. (2014). Skewer: a fast and accurate adapter trimmer for next-generation sequencing paired-end reads. BMC Bioinformatics 15:182. 10.1186/1471-2105-15-182 24925680 PMC4074385

[B33] Kaleta C. Schäuble S. Rinas U. Schuster S. (2013). Metabolic costs of amino acid and protein production in *Escherichia coli*. Biotechnol. J. 8, 1105–1114. 10.1002/biot.201200267 23744758

[B34] Kotlowski R. Bernstein C. N. Sepehri S. Krause D. O. (2007). High prevalence of escherichia coli belonging to the B2+D phylogenetic group in inflammatory bowel disease. Gut 56, 669–675. 10.1136/gut.2006.099796 17028128 PMC1942160

[B35] Langdon A. Crook N. Dantas G. (2016). The effects of antibiotics on the microbiome throughout development and alternative approaches for therapeutic modulation. Genome Med. 8:39. 10.1186/s13073-016-0294-z 27074706 PMC4831151

[B36] Langmead B. Salzberg S. L. (2012). Fast gapped-read alignment with bowtie 2. Nat. Methods 9, 357–359. 10.1038/nmeth.1923 22388286 PMC3322381

[B37] Luo C. Knight R. Siljander H. Knip M. Xavier R. J. Gevers D. (2015). ConStrains identifies microbial strains in metagenomic datasets. Nat. Biotechnol. 33, 1045–1052. 10.1038/nbt.3319 26344404 PMC4676274

[B38] Martin M. (2011). Cutadapt removes adapter sequences from high-throughput sequencing reads. EMBnet. J. 17, 10–12. 10.14806/ej.17.1.200

[B39] Martinez-Jéhanne V. du Merle L. Bernier-Fébreau C. Usein C. Gassama-Sow A. Wane A.-A.. (2009). Role of deoxyribose catabolism in colonization of the murine intestine by pathogenic *Escherichia coli* strains. Infect. Immun. 77, 1442–1450. 10.1128/IAI.01039-08 19168744 PMC2663165

[B40] Martinez-Medina M. Garcia-Gil L. J. (2014). *Escherichia coli* in chronic inflammatory bowel diseases: an update on adherent invasive escherichia coli pathogenicity. World J. Gastrointest. Pathophysiol. 5, 213–227. 10.4291/wjgp.v5.i3.213 25133024 PMC4133521

[B41] Martinez-Medina M. Mora A. Blanco M. López C. Alonso M. P. Bonacorsi S.. (2009). Similarity and divergence among adherent-invasive *Escherichia coli* and extraintestinal pathogenic *E. coli* strains. J. Clin. Microbiol. 47, 3968–3979. 10.1128/JCM.01484-09 19828750 PMC2786640

[B42] Matsuoka K. Kanai T. (2015). The gut microbiota and inflammatory bowel disease. Semin. Immunopathol. 37, 47–55. 10.1007/s00281-014-0454-4 25420450 PMC4281375

[B43] mca (2018). mca. Available online at: https://pypi.org/project/mca/ (Accessed May 4, 2018).

[B44] McCloskey D. Palsson B. Ø. Feist A. M. (2013). Basic and applied uses of genome–scale metabolic network reconstructions of *Escherichia coli*. Mol. Syst. Biol. 9:661. 10.1038/msb.2013.18 23632383 PMC3658273

[B45] McGinnis S. Madden T. L. (2004). BLAST: at the core of a powerful and diverse set of sequence analysis tools. Nucleic Acids Res. 32, W20–W25. 10.1093/nar/gkh435 15215342 PMC441573

[B46] Miquel S. Peyretaillade E. Claret L. de Vallée A. Dossat C. Vacherie B.. (2010). Complete genome sequence of crohn's disease-associated adherent-invasive *E. coli* strain LF82. PLoS ONE 5:e12714. 10.1371/journal.pone.0012714 20862302 PMC2941450

[B47] Monk J. Nogales J. Palsson B. O. (2014). Optimizing genome-scale network reconstructions. Nat. Biotechnol. 32, 447–452. 10.1038/nbt.2870 24811519

[B48] Monk J. M. Charusanti P. Aziz R. K. Lerman J. A. Premyodhin N. Orth J. D.. (2013). Genome-scale metabolic reconstructions of multiple escherichia coli strains highlight strain-specific adaptations to nutritional environments. Proc. Natl. Acad. Sci. U.S.A. 110, 20338–20343. 10.1073/pnas.1307797110 24277855 PMC3864276

[B49] Monk J. M. Lloyd C. J. Brunk E. Mih N. Sastry A. King Z.. (2017). iML1515, a knowledgebase that computes escherichia coli traits. Nat. Biotechnol. 35, 904–908. 10.1038/nbt.3956 29020004 PMC6521705

[B50] Morgan X. C. Tickle T. L. Sokol H. Gevers D. Devaney K. L. Ward D. V.. (2012). Dysfunction of the intestinal microbiome in inflammatory bowel disease and treatment. Genome Biol. 13:R79. 10.1186/gb-2012-13-9-r79 23013615 PMC3506950

[B51] Mosli M. H. Zou G. Garg S. K. Feagan S. G. MacDonald J. K. Chande N.. (2015). C-Reactive protein, fecal calprotectin, and stool lactoferrin for detection of endoscopic activity in symptomatic inflammatory bowel disease patients: a systematic review and Meta-Analysis. Am. J. Gastroenterol. 110, 802–819. quiz: 820. 10.1038/ajg.2015.120 25964225

[B52] Mukhopadhya I. Hansen R. El-Omar E. M. Hold G. L. (2012). IBD—what role do proteobacteria play? Nat. Rev. Gastroenterol. Hepatol. 9:219. 10.1038/nrgastro.2012.14 22349170

[B53] Nash J. H. Villegas A. Kropinski A. M. Aguilar-Valenzuela R. Konczy P. Mascarenhas M.. (2010). Genome sequence of adherent-invasive *Escherichia coli* and comparative genomic analysis with other *E. coli* pathotypes. BMC Genomics 11:667. 10.1186/1471-2164-11-667 21108814 PMC3091784

[B54] Nayfach S. Rodriguez-Mueller B. Garud N. Pollard K. S. (2016). An integrated metagenomics pipeline for strain profiling reveals novel patterns of bacterial transmission and biogeography. Genome Res. 26, 1612–1625. 10.1101/gr.201863.115 27803195 PMC5088602

[B55] Negroni A. Costanzo M. Vitali R. Superti F. Bertuccini L. Tinari A.. (2012). Characterization of adherent-invasive *Escherichia coli* isolated from pediatric patients with inflammatory bowel disease. Inflamm. Bowel Dis. 18, 913–924. 10.1002/ibd.21899 21994005

[B56] Neidhardt F. C. Curtiss R. (1999). Escherichia coli and Salmonella: Cellular and Molecular Biology, Vol. 2 Washington, DC: ASM Press.

[B57] Ni J. Wu G. D. Albenberg L. Tomov V. T. (2017). Gut microbiota and IBD: causation or correlation? Nat. Rev. Gastroenterol. Hepatol. 14, 573–584. 10.1038/nrgastro.2017.88 28743984 PMC5880536

[B58] O'Brien C. L. Bringer M. A. Holt K. E. Gordon D. M. Dubois A. L. Barnich N.. (2016). Comparative genomics of crohn's disease-associated adherent-invasive *Escherichia coli*. Gut 66, 1382–1389. 10.1136/gutjnl-2015-311059 27196580

[B59] Page A. J. Cummins C. A. Hunt M. Wong V. K. Reuter S. Holden M. T. G.. (2015). Roary: rapid large-scale prokaryote pan genome analysis. Bioinformatics 31, 3691–3693. 10.1093/bioinformatics/btv421 26198102 PMC4817141

[B60] Palmela C. Chevarin C. Xu Z. Torres J. Sevrin G. Hirten R.. (2017). Adherent-invasive *Escherichia coli* in inflammatory bowel disease. Gut 67, 574–587. 10.1136/gutjnl-2017-314903 29141957

[B61] Parks D. H. Imelfort M. Skennerton C. T. Hugenholtz P. Tyson G. W. (2015). CheckM: assessing the quality of microbial genomes recovered from isolates, single cells, and metagenomes. Genome Res. 25, 1043–1055. 10.1101/gr.186072.114 25977477 PMC4484387

[B62] Pascal V. Pozuelo M. Borruel N. Casellas F. Campos D. Santiago A.. (2017). A microbial signature for crohn's disease. Gut 66, 813–822. 10.1136/gutjnl-2016-313235 28179361 PMC5531220

[B63] Petersen A. M. Nielsen E. M. Litrup E. Brynskov J. Mirsepasi H. Krogfelt K. A. (2009). A phylogenetic group of *Escherichia coli* associated with active left-sided inflammatory bowel disease. BMC Microbiol. 9:171. 10.1186/1471-2180-9-171 19695087 PMC2736970

[B64] Quince C. Delmont T. O. Raguideau S. Alneberg J. Darling A. E. Collins G.. (2017). DESMAN: a new tool for *de novo* extraction of strains from metagenomes. Genome Biol. 18:181. 10.1186/s13059-017-1309-9 28934976 PMC5607848

[B65] Rasko D. A. Rosovitz M. J. Myers G. S. A. Mongodin E. F. Fricke W. F. Gajer P.. (2008). The pangenome structure of *Escherichia coli*: comparative genomic analysis of *E. coli* commensal and pathogenic isolates. J. Bacteriol. 190, 6881–6893. 10.1128/JB.00619-08 18676672 PMC2566221

[B66] Ravcheev D. A. Thiele I. (2017). Comparative genomic analysis of the human gut microbiome reveals a broad distribution of metabolic pathways for the degradation of Host-Synthetized mucin glycans and utilization of Mucin-Derived monosaccharides. Front. Genet. 8:111. 10.3389/fgene.2017.00111 28912798 PMC5583593

[B67] Rhodes J. M. (2007). The role of *Escherichia coli* in inflammatory bowel disease. Gut 56, 610–612. 10.1136/gut.2006.111872 17440180 PMC1942130

[B68] Ruan Z. Feng Y. (2016). BacWGSTdb, a database for genotyping and source tracking bacterial pathogens. Nucleic Acids Res. 44, D682–D687. 10.1093/nar/gkv1004 26433226 PMC4702769

[B69] Sasaki M. Sitaraman S. V. Babbin B. A. Gerner-Smidt P. Ribot E. M. Garrett N.. (2007). Invasive *Escherichia coli* are a feature of crohn's disease. Lab. Invest. 87, 1042–1054. 10.1038/labinvest.3700661 17660846

[B70] Schirmer M. Franzosa E. A. Lloyd-Price J. McIver L. J. Schwager R. Poon T. W.. (2018). Dynamics of metatranscription in the inflammatory bowel disease gut microbiome. Nat. Microbiol. 3, 337–346. 10.1038/s41564-017-0089-z 29311644 PMC6131705

[B71] sci (2018). scikit-bio. Available online at: http://scikit-bio.org/ (Accessed May 3, 2018).

[B72] Seemann T. (2014). Prokka: rapid prokaryotic genome annotation. Bioinformatics 30, 2068–2069. 10.1093/bioinformatics/btu153 24642063

[B73] Segata N. Bornigen D. Morgan X. C. Huttenhower C. (2013). PhyloPhlAn is a new method for improved phylogenetic and taxonomic placement of microbes. Nat. Commun. 4:2304. 10.1038/ncomms3304 23942190 PMC3760377

[B74] Sharpton T. Lyalina S. Luong J. Pham J. Deal E. M. Armour C.. (2017). Development of inflammatory bowel disease is linked to a longitudinal restructuring of the gut metagenome in mice. mSystems 2, e00036–17. 10.1128/mSystems.00036-17 28904997 PMC5585689

[B75] Snipen L. Almøy T. Ussery D. W. (2009). Microbial comparative pan-genomics using binomial mixture models. BMC Genomics 10:385. 10.1186/1471-2164-10-385 19691844 PMC2907702

[B76] The UniProt Consortium (2017). UniProt: the universal protein knowledgebase. Nucleic Acids Res. 45, D158–D169. 10.1093/nar/gkw1099 27899622 PMC5210571

[B77] Thiele I. Palsson B. Ø. (2010). A protocol for generating a high-quality genome-scale metabolic reconstruction. Nat. Protoc. 5, 93–121. 10.1038/nprot.2009.203 20057383 PMC3125167

[B78] Truong D. T. Tett A. Pasolli E. Huttenhower C. Segata N. (2017). Microbial strain-level population structure and genetic diversity from metagenomes. Genome Res. 27, 626–638. 10.1101/gr.216242.116 28167665 PMC5378180

[B79] Vazeille E. Chassaing B. Buisson A. Dubois A. de Vallée A. Billard E.. (2016). GipA factor supports colonization of peyer's patches by crohn's disease-associated *Escherichia coli*. Inflamm. Bowel Dis. 22, 68–81. 10.1097/MIB.0000000000000609 26512715

[B80] Vejborg R. M. Hancock V. Petersen A. M. Krogfelt K. A. Klemm P. (2011). Comparative genomics of *Escherichia coli* isolated from patients with inflammatory bowel disease. BMC Genomics 12:316. 10.1186/1471-2164-12-316 21676223 PMC3155842

[B81] Wu S. Li W. Smarr L. Nelson K. Yooseph S. Torralba M. (2013). Large memory high performance computing enables comparison across human gut microbiome of patients with autoimmune diseases and healthy subjects, in Proceedings of the Conference on Extreme Science and Engineering Discovery Environment: Gateway to Discovery (San Diego, CA: ACM), 25.

[B82] Wu Y. W. Simmons B. A. Singer S. W. (2016). MaxBin 2.0: an automated binning algorithm to recover genomes from multiple metagenomic datasets. Bioinformatics 32, 605–607. 10.1093/bioinformatics/btv638 26515820

[B83] Zhang Y. Rowehl L. Krumsiek J. M. Orner E. P. Shaikh N. Tarr P. I. (2015). Identification of candidate adherent-invasive *E. coli* signature transcripts by genomic/transcriptomic analysis. PLoS ONE 10:e0130902 10.1371/journal.pone.0130902 26125937 PMC4509574

