# Supplementary material for: Metagenomics-Based, Strain-Level Analysis of Escherichia coli From a Time-Series of Microbiome Samples From a Crohn's Disease Patient
Source: Front Microbiol. 2018 Oct 30;9:2559. doi: 10.3389/fmicb.2018.02559 (PMC6218438; doi:10.3389/fmicb.2018.02559)
Supplement: Supplementary file 1 [file Data_Sheet_1.PDF]

Supplementary text

Identification of bacteriophages from metagenomic data

During the preliminary analysis on the metagenomics data, we processed the data and identified microbial species using the computational analysis described in a previous publication (Wu et al., 2013). We mapped high-quality reads to a reference database of curated microbial genomes using fr-hit (Niu et al., 2011), and found that two enterobacteriophages K1E and K1-5 may be present in TP1 at high abundance while absent from other samples.

To confirm the presence of the two phages in TP1, we mapped the high-quality reads from TP1 to the genome sequences of these phages (accessions: NC_007637.1 and NC_008152.1) using Bowtie2 (Langmead and Salzberg, 2012). The result suggests that the two phages are indeed present in TP1, as the majority of the phage genomes is covered, and at a relative constant depth (see Fig. S1). The overall alignment rates reported by Bowtie2 to K1E and K1-5 in the TP1 sample sequenced are 0.02% and 0.03%, respectively. The two phages were not captured by MetaPhlAn2 due to the limitation of its reference database.

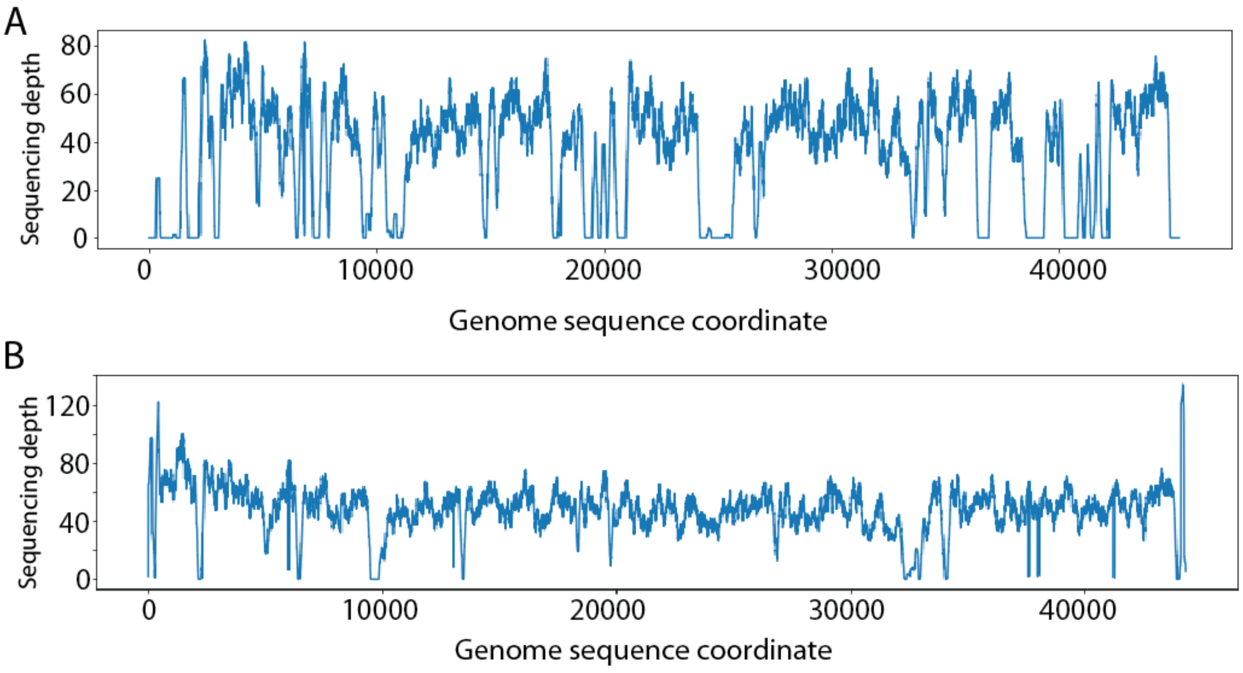

Figure S1: Coverage of enterobacteriophages K1E (A) and K1-5 (B) in sample TP1.

CG1MAC and 3_2_53FAA do not display AIEC phenotypes

Adhesion of bacteria on Caco2 epithelial cells at an MOI 1:100 for strain 3_2_53FAA was on average 22.7±11.4 bacteria/cell, and for CG1MAC 9.32±3.6 bacteria/cell; similar to AIEC strain O83 which had 5.78±2.2 bacteria/cell which is in direct contrast with LF82 strain 3.34±1 bacteria/cell. However, the lack of surface adhered bacteria in the LF82 strain is partially compensated by the number of bacteria found internalized in the Caco2 cells. In the invasion assay the strain L82 had 0.1±0.05 bacteria/cell, tenfold higher than the closely related AIEC strain O83 which had 0.009±0.007 bacteria/cell, and both strains 3_2_53FAA and CG1MAC had 0.004±0.002 bacterial/cell, similar levels found in non-pathogenic control strains F18 (commensal *E. coli*) 0.004 ±0.003 and HB101 (lab strain) 0.003±0.001 bacteria/cell.

Survival of *E. coli* in macrophages is considered a key characteristic of AIEC strains. The survival rate of the well characterized AIEC strain LF82 in THP-1 macrophages was 133.5% after 24 hours incubation, strain O83 survival rate was 73.5%, 3_2_53FAA was 30.7% and CG1MAC was 40.1%; control strains F18 survival rate was 32.6% and HB101 was 27.8%. The low survival rate of 3_2_53FAA and CG1MAC are comparable to the control strains indicating that they do not fit the classical characterization of AIEC strains.

Comparative analysis of CG1MAC and 3_2_53FAA

E. coli strain 3_2_53FAA was previously isolated from a patient with Crohn's disease, in Calgary, Canada and was shown to be closely related to an *E. coli* strain of interest identified bioinformatically in a Crohn's disease patient in San Diego, USA. 3_2_53FAA was first isolated from the inflamed descending colon of a 52-year-old male CD patient, and sequenced by The Broad Institute as part of the HMP reference genome collection. It has a 5,158,378 bp genome and 5,078 coding regions. Comparative genome analysis showed that the San Diego patient's *E. coli* sequence shared 4837/4916 ORFs with 3_2_53FAA. Similar to CG1MAC, 3_2_53FAA also belongs to phylogroup B2, and is evolutionarily closely related to AIEC and ExPEC strains. We determined the serotype of 3_2_53FAA to be O1:H6, which is different from the serotype of CG1MAC (O2:H7). Additionally, adhesion and invasion assays showed that 3_2_53FAA also does not display AIEC characteristics - it is able to adhere well to epithelial cell Caco-2 but does not invade it.

Comparative GEMs analysis also showed similarity in metabolic functions between 3_2_FAA and CG1MAC. We built the draft model of 3_2_53FAA based on its genome,

and simulated growth on different nutrient sources. GEMs of CG1MAC and 3_2_53FAA predict that these two strains have the same growth capability on more than 300 nutrient sources tested. This result suggests that 3_2_53FAA and CG1MAC share similar metabolic functions and potentially colonize common nutrient niches.

Growth experiments

We then experimentally validated several GEM predictions with growth assays for K-12 and CG1MAC. A total of 15 nutrient sources were tested and the comparison with predicted model simulation is shown in Table S4. Out of the 30 results, the GEMs correctly predicted 21 growth capabilities. Most of the inaccurate predictions were false positives, for which growth was predicted but was not seen experimentally. The inaccurate predictions could potentially be due to the assumption of optimal growth performance of the strain, while the growth experiments only lasted 48 hrs, which might have represented insufficient time for the strains to completely adapt to a new nutrient source (Guzmán et al., 2015).

Supplementary figures

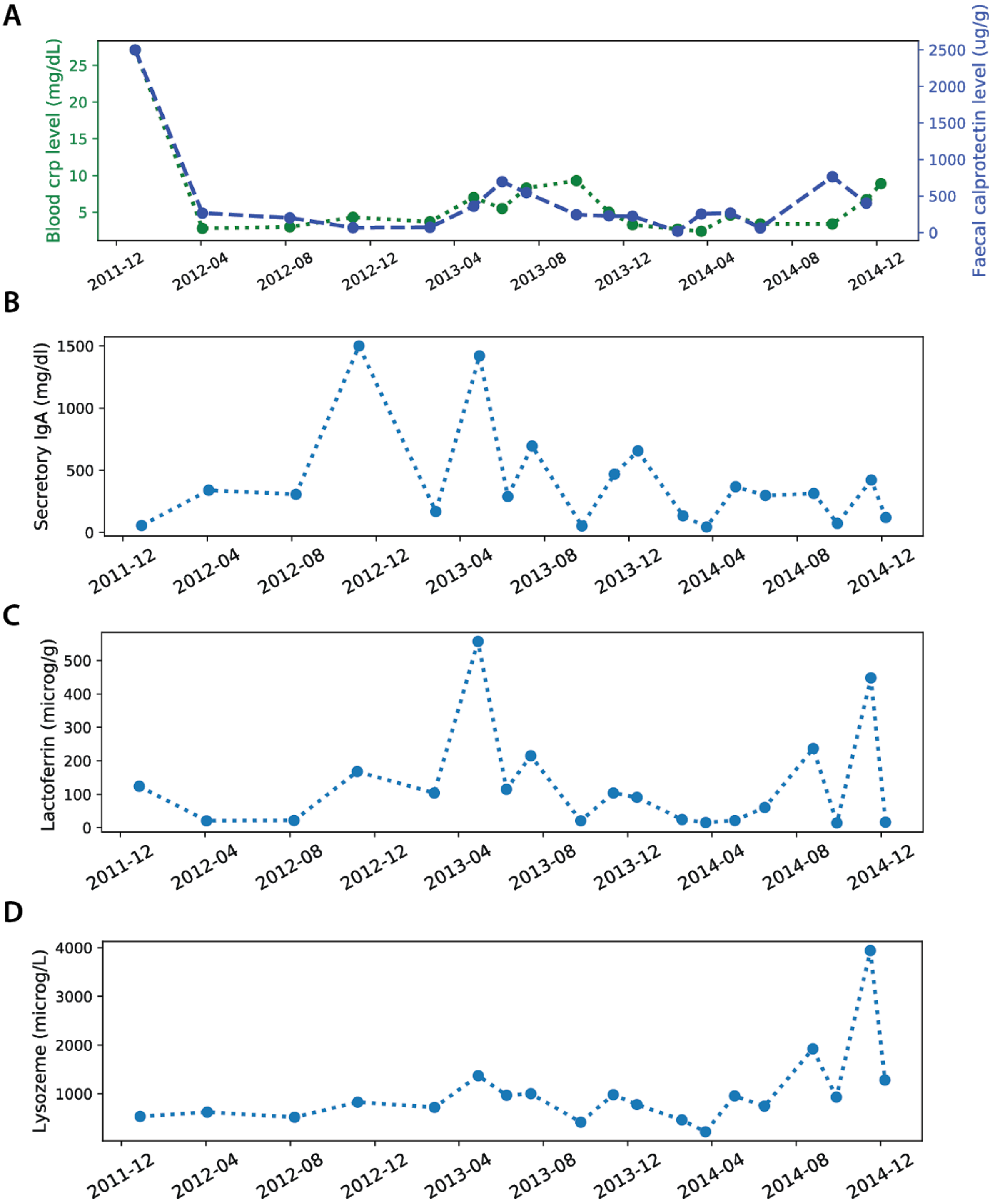

Figure S2: Biomarkers measured over three years. **A)** Faecal calprotectin level displayed similar trend to blood CRP level. B) Secretory IgA level. C) Lactoferrin level. D) Lysozyme level

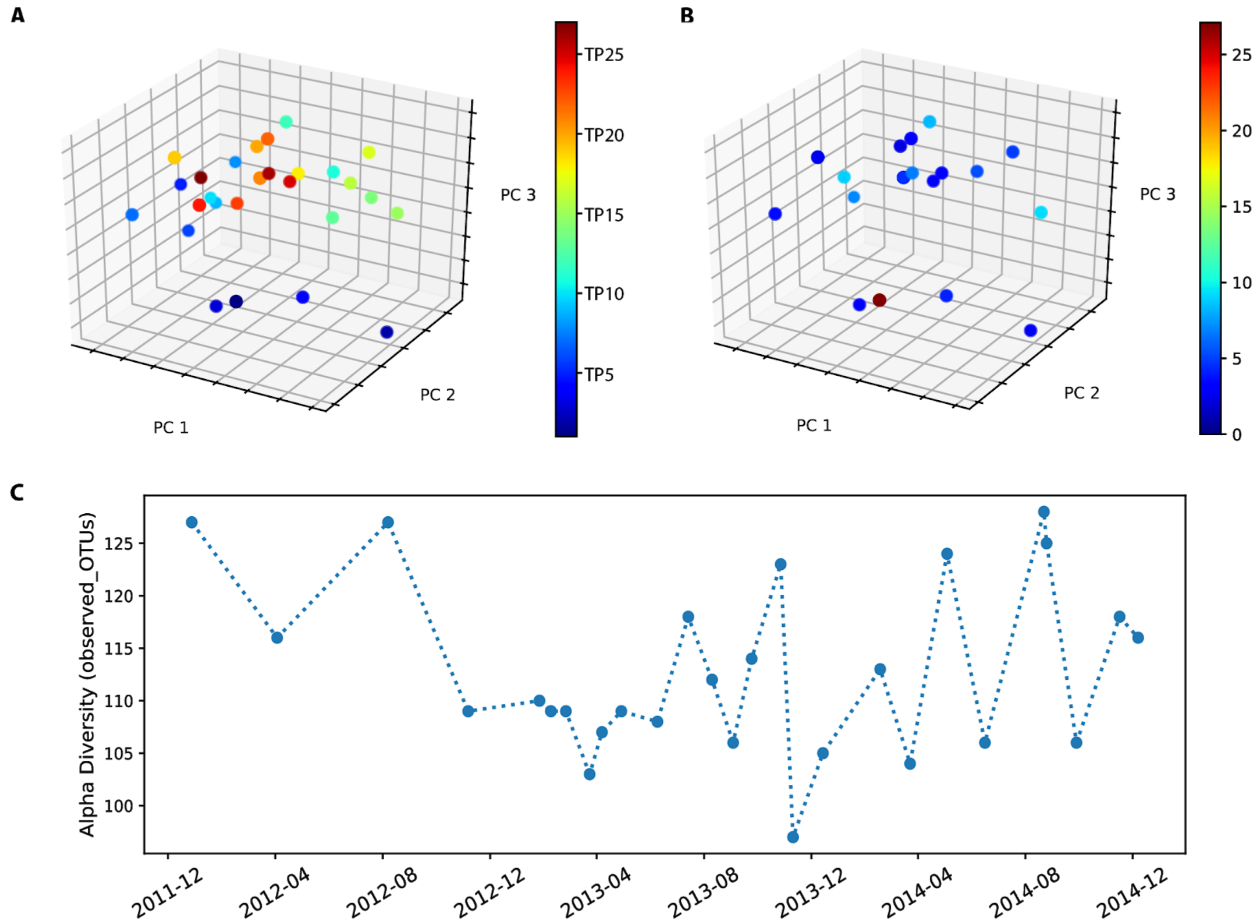

Figure S3: Principal coordinates analysis of beta diversity of 21 samples. A) 21 samples labeled by time the sample was collected. B) 18 samples labeled by blood CRP level. C) Alpha diversity calculated over time.

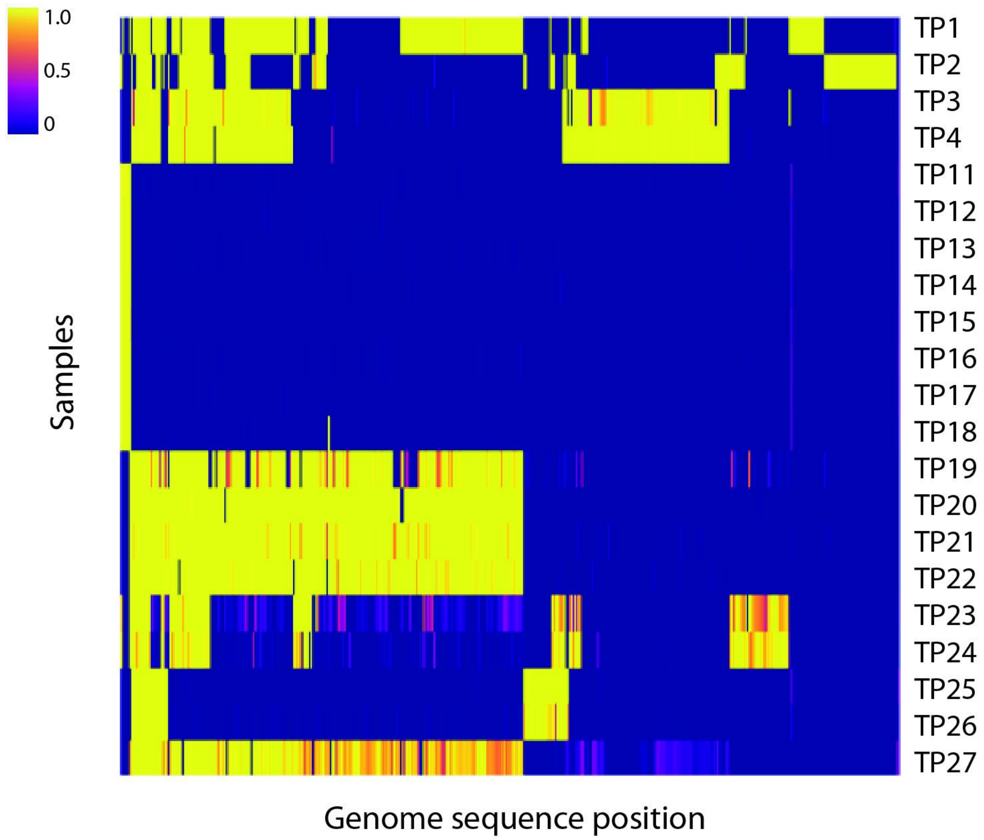

Figure S4. Heatmap of MIDAS genome-wide SNV frequencies for *E.coli* population across metagenomic samples. Rows correspond to samples and columns correspond to positions of SNVs detected by MIDAS (clustered by their frequencies across samples).

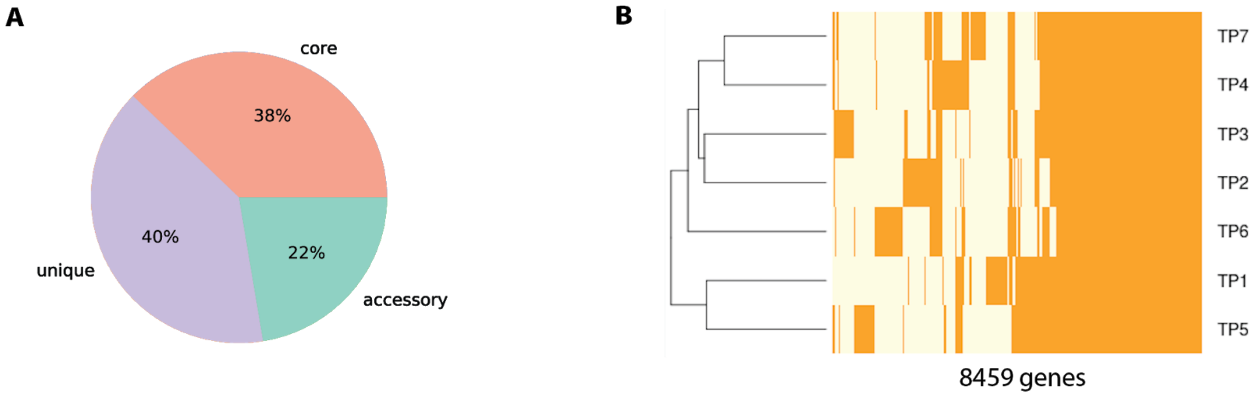

Figure S5: Pan genome of 7 dominant strains. A) Distribution of core, accessory and unique genes. B) Presence/absence of genes are shown in the heatmap.

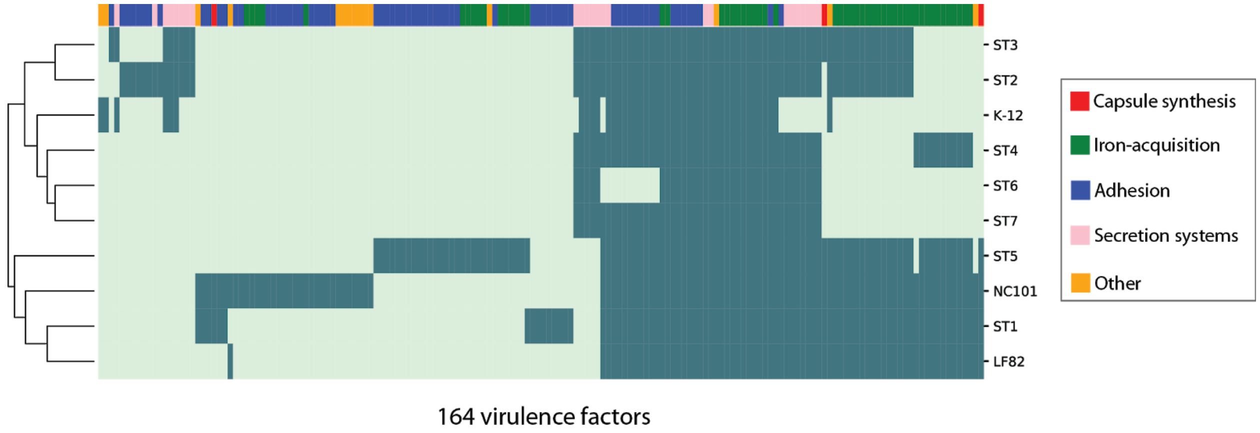

Figure S6: Presence/absence of 164 virulence factors from VFDB in the dominant *E. coli* assemblies and reference strains.

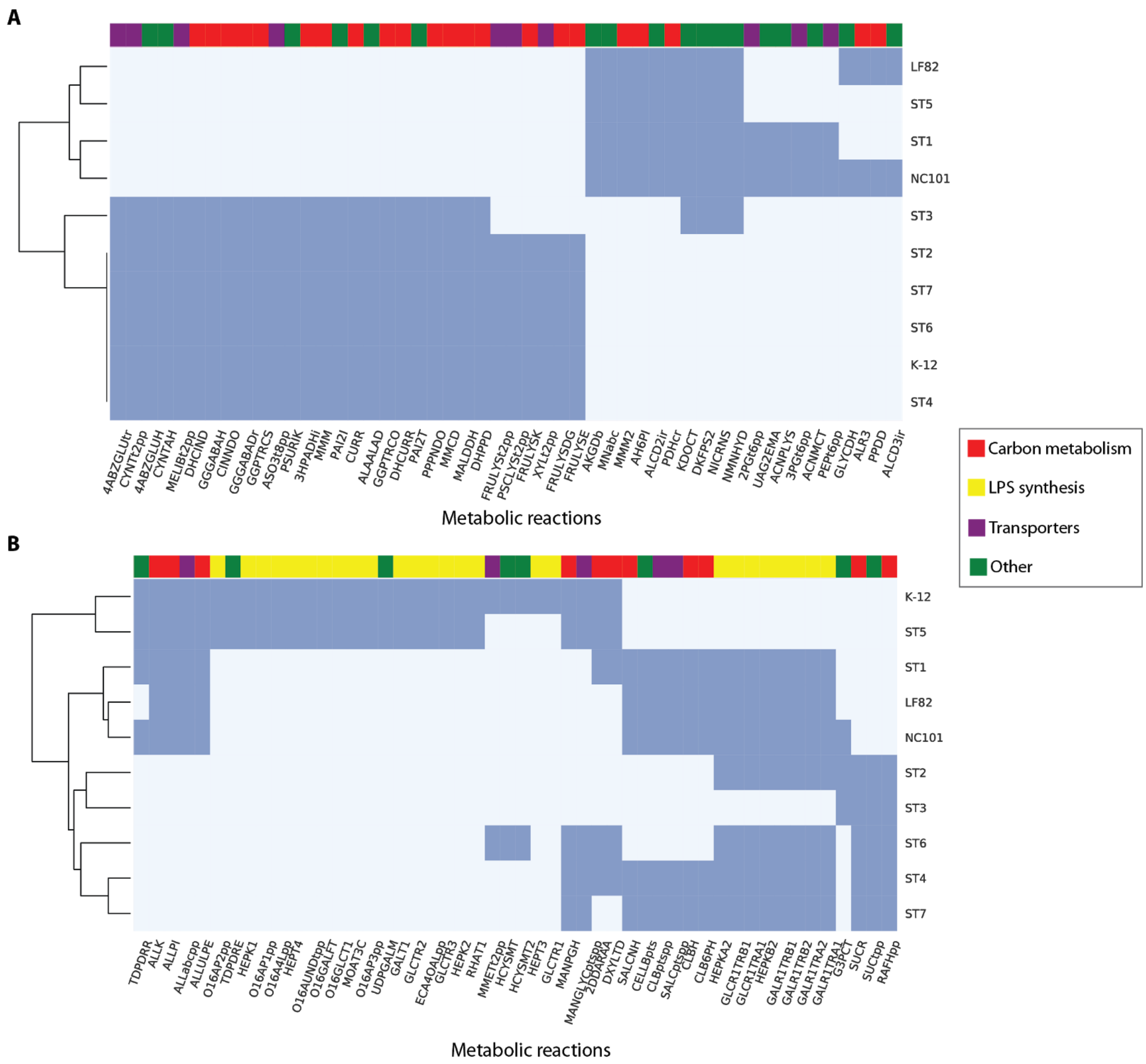

Figure S7: Presence/absence of important reactions identified in factor 1 and 2 in MCA. Functions of these reactions are color labeled. A) Distribution of top 50 reactions in factor 1 in 10 strains. B) Distribution of top 50 reactions in factor 2 in 10 strains.

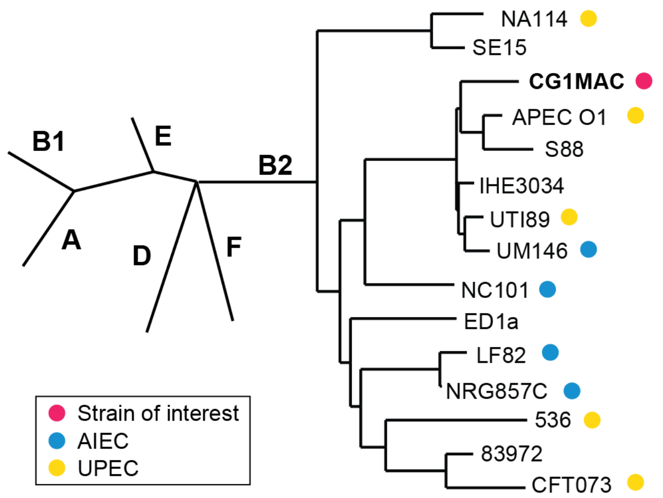

Figure S8: The phylogenetic position of CG1MAC suggests that it is closely related to AIEC and uropathogenic (UPEC) strains in phylogroup B2.

References

Guzmán, G. I., Utrilla, J., Nurk, S., Brunk, E., Monk, J. M., Ebrahim, A., et al. (2015). Model-driven discovery of underground metabolic functions in Escherichia coli. *Proc. Natl. Acad. Sci. U. S. A.* 112, 929–934.

Langmead, B., and Salzberg, S. L. (2012). Fast gapped-read alignment with Bowtie 2. *Nat. Methods* 9, 357–359.

Niu, B., Zhu, Z., Fu, L., Wu, S., and Li, W. (2011). FR-HIT, a very fast program to recruit metagenomic reads to homologous reference genomes. *Bioinformatics* 27, 1704–1705.

Wu, S., Li, W., Smarr, L., Nelson, K., Yooseph, S., and Torralba, M. (2013). Large memory high performance computing enables comparison across human gut microbiome of patients with autoimmune diseases and healthy subjects. in *Proceedings of the Conference on Extreme Science and Engineering Discovery Environment: Gateway to Discovery* (ACM), 25.
